# Nanoparticle-Enabled Immune-Dose Shaping in Breast Cancer Radiotherapy: Reconciling Tumor Immunogenicity with Systemic Immune Preservation

**DOI:** 10.3390/ijms27198928

**Published:** 2026-10-08

**Authors:** Sorinel Lunca, Stefan Morarasu, Gabriel Mihail Dimofte

**Affiliations:** 1 Grigore T Popa University of Medicine and Pharmacy, 700115 Iasi, Romania; sorinel.lunca@umfiasi.ro (S.L.); mihail.dimofte@umfiasi.ro (G.M.D.); 2 2nd Department of Surgical Oncology, Regional Institute of Oncology, 700483 Iasi, Romania

**Keywords:** breast cancer, radiotherapy, nanoparticles, radioenhancement, radiation-induced lymphopenia, immune-dose shaping, tumor immunogenicity

## Abstract

Radiotherapy can simultaneously promote tumor immunogenicity and damage the systemic immune compartments required to sustain antitumor responses. In breast cancer, radiation-induced lymphopenia is influenced by treatment geometry, irradiated volume, low-dose exposure, marrow and lymphoid irradiation, fractionation, and delivery characteristics, while nanoparticle radioenhancement can amplify tumor response through physical, redox, DNA-repair, and immune mechanisms. This hypothesis-driven translational review examines whether nanoparticle-generated biological amplification could be redirected from tumor intensification toward immune-dose shaping while preserving tumor control and immunogenicity. The evidence supports seven interacting dimensions of potential exchange: physical dose, volume/spatial exposure, distributed low-dose exposure, marrow dose, geometry/modality, fractionation, and temporal/delivery exposure. Formal nanoparticle-enabled dose exchange remains limited, with the clearest breast-specific evidence confined to in vitro isoeffect analysis; no study has yet demonstrated preserved tumor isoeffect and immunogenicity together with reduced multicompartment immune injury after nanoparticle-enabled exposure reduction. We, therefore, propose a dual-endpoint framework integrating tumor control, immunogenicity, multicompartment immune preservation, dosimetry, and nanoparticle biodistribution, together with a staged translational pathway from quantitative preclinical exchange studies to comparative clinical testing. This strategy remains hypothesis-generating, with triple-negative breast cancer representing a particularly informative initial setting.

## 1. Introduction

Radiotherapy (RT) is a cornerstone of breast cancer treatment, but its effects extend beyond direct tumor-cell killing. Ionizing radiation can promote immunogenic cell death (ICD), expose calreticulin, release ATP and HMGB1, generate cytosolic double-stranded DNA, and activate the cGAS–STING/type I interferon axis, supporting dendritic-cell maturation, antigen presentation, and recruitment of tumor-reactive T cells [1,2,3,4]. These responses depend strongly on dose, fractionation, and tumor biology [3,5,6,7,8].

Nanoparticle radioenhancement can amplify radiation effects through increased energy deposition, reactive oxygen species generation, hypoxia/redox modulation, DNA-repair interference, ferroptosis, or direct immune activation [9,10,11,12]. Hafnium oxide NBTXR3 increases DNA damage and cGAS–STING signaling and can broaden tumor immunogenicity and T-cell receptor repertoire [13,14]. Breast-specific platforms using manganese, platinum, gadolinium, lutetium, and gold nanomaterials similarly enhance ICD, dendritic-cell maturation, macrophage polarization, CD8+ recruitment, and, in selected models, distant tumor control and immune memory [10,12,15,16,17,18,19,20,21].

The same radiation exposure, however, can damage the immune system required to sustain these effects. Radiation-induced lymphopenia (RIL) in breast cancer is associated with treatment volume, regional nodal irradiation, low-dose exposure, effective dose to immune cells (EDIC), fractionation, technique, and marrow exposure [22,23,24,25,26]. Severe or persistent lymphopenia has been associated with poorer outcomes, although whether RIL is causal or reflects treatment intensity and baseline vulnerability remains unresolved [27,28,29,30,31]. RIL is also unlikely to arise from circulating-blood irradiation alone: dynamic models support repeated blood-pool exposure across fractions, clinical studies implicate hematopoietic marrow, and mechanistic evidence identifies tumor-draining lymph nodes as reservoirs contributing activated CD8+ T cells to irradiated tumors [25,26,32,33,34,35,36,37]. The immune system is, therefore, better considered a distributed organ at risk encompassing blood, lymphoid tissue, marrow, and tumor-associated immune cells [38,39,40].

This paradox may be particularly relevant in triple-negative breast cancer (TNBC), where tumor immune infiltration is prognostically important [41] and higher EDIC has been associated with poorer event-free survival in an exploratory cohort [42]. TNBC is also disproportionately represented in preclinical nanoparticle–radioimmunotherapy models, making it a logical initial testing context without implying exclusivity [21].

Importantly, immune-relevant radiation exposure is modifiable. Proton therapy, breath hold, and positioning can reduce modeled EDIC; fractionation and restricted-volume approaches can reduce lymphocyte exposure or depletion [29,43,44,45]; and reduced-volume irradiation can preserve lymphocytes while retaining cGAS–STING activation and tumor control [46,47]. FLASH adds a temporal dimension: ex vivo data show reduced chromosomal injury at ultra-high dose rates, while preclinical and computational studies demonstrate treatment-dependent immune effects that remain compartment-dependent [48,49,50,51]. Thus, immune injury reflects the spatial and temporal distribution of exposure, not prescription dose alone.

This creates an opportunity for nanoparticle radioenhancement distinct from tumor intensification. Greater tumor effect at an unchanged dose demonstrates radioenhancement, not dose exchange. True exchange requires a lower-dose NP + RT regimen to reproduce a predefined tumor effect achieved with higher-dose RT alone. Breast-specific in vitro dose–response and isoeffect studies show that a quantifiable exchange margin is possible, and computational and cross-tumor studies provide additional proof of concept, but clean in vivo demonstration in breast cancer remains lacking [11,52,53,54]. Clinical NBTXR3 studies establish a meaningful biological amplification margin, but this has so far been used to intensify tumor response rather than reduce physical exposure [55,56,57].

Dose reduction alone may also provide limited immune benefit if irradiated volume, distributed low-dose exposure, marrow or nodal exposure, and fraction number remain unchanged. We, therefore, propose nanoparticle-enabled immune-dose shaping: using nanoparticle-generated tumor biological amplification as a potentially exchangeable margin to reduce selected components of physical, spatial, or temporal radiation exposure while preserving tumor control and immunogenicity.

The evidence gap is specific. Nanoparticle studies generally assess tumor radiosensitization and immunogenicity without measuring systemic immune preservation, whereas breast RIL and dosimetry studies quantify lymphocyte depletion or immune exposure without establishing preservation of tumor immunogenicity. No study identified in this evidence set has combined nanoparticle-enabled reductions in immune-relevant radiation exposure, preserved tumor isoeffect, maintained immunogenicity, and reduced multicompartment immune injury within the same breast-cancer experiment or cohort.

This review, therefore, integrates clinical breast RIL and dosimetry evidence with nanoparticle radioenhancement, immunogenic RT, and geometric/temporal sparing; distinguishes same-dose enhancement from genuine dose exchange; and proposes a dual tumor–immune framework evaluating tumor control and immunogenicity alongside injury to circulating, lymphoid, and hematopoietic compartments. The result is not an established therapeutic strategy but a falsifiable translational hypothesis. Although individual components are supported by clinical, preclinical, and computational evidence, the integrated concept of nanoparticle-enabled immune-dose shaping has not yet been experimentally demonstrated. This review, therefore, proposes a proof-of-concept research framework for investigating whether nanoparticle-generated biological amplification can be redistributed to reduce immune-relevant radiation exposure while preserving tumor control and immunogenicity.

**Figure 1:**
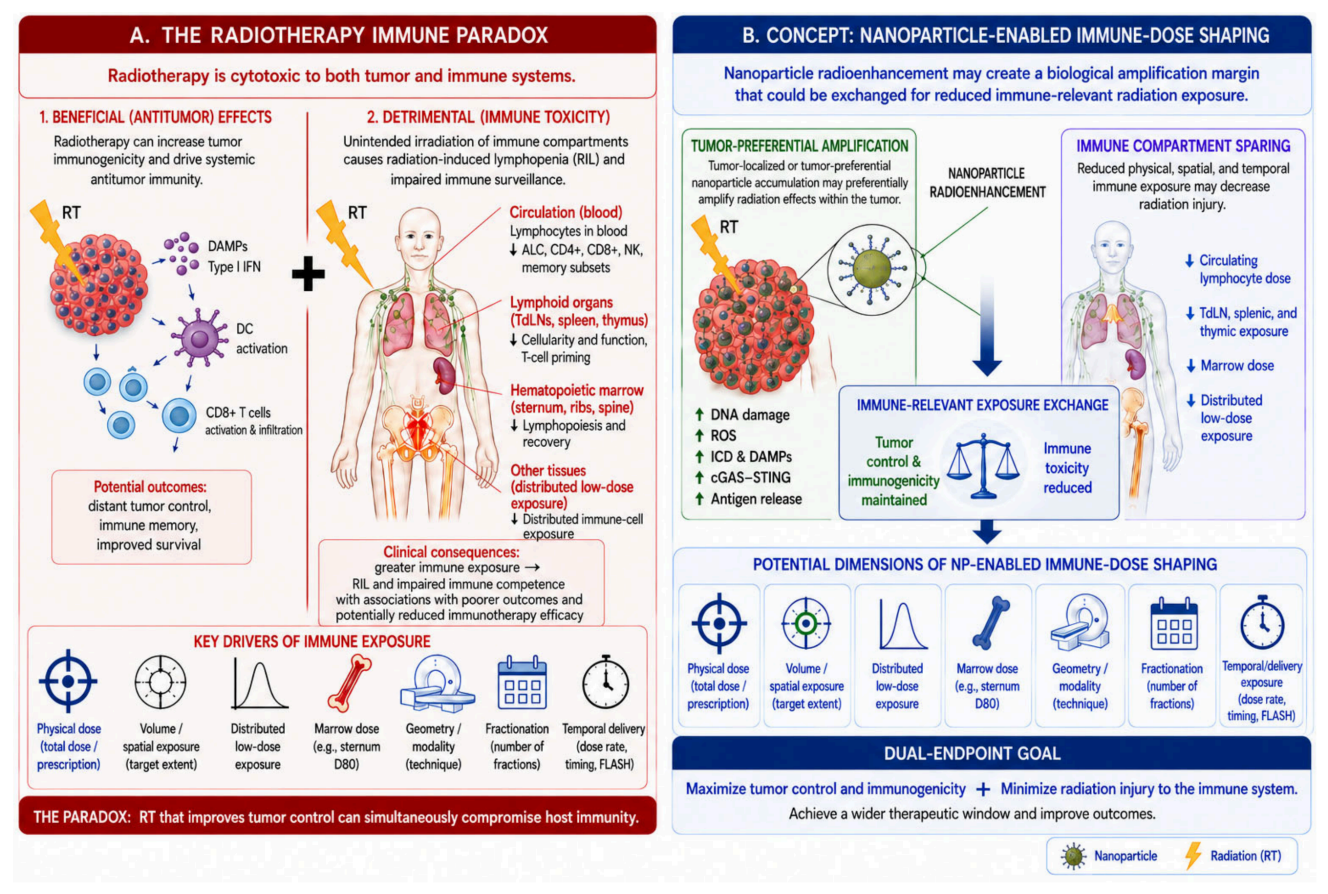
**The radiotherapy immune paradox and the concept of nanoparticle-enabled immune-dose shaping.** Radiotherapy can simultaneously enhance tumor immunogenicity through DNA damage, damage-associated molecular pattern release, cGAS–STING/type I interferon signaling, dendritic-cell activation, and T-cell recruitment, while also injuring circulating lymphocytes, lymphoid organs, and hematopoietic marrow. The resulting immune cost is influenced by treated volume and spatial extent, distributed low-dose exposure, marrow dose, treatment geometry and modality, fractionation, and temporal delivery. Nanoparticle radioenhancement may create a biological amplification margin that could be exploited to reduce physical, spatial, or temporal immune exposure while preserving tumor control and immunogenicity. These approaches must remain within oncologic target-coverage constraints. The dual objective is to maintain tumor efficacy and immunogenicity while minimizing radiation injury to the immune system. **RT**, radiotherapy; **DAMPs**, damage-associated molecular patterns; **IFN**, interferon; **DC**, dendritic cell; **TdLN**, tumor-draining lymph node; **ALC**, absolute lymphocyte count; **ICD**, immunogenic cell death; **NK**, natural killer; **ROS**, reactive oxygen species; **cGAS–STING**, cyclic GMP–AMP synthase–stimulator of interferon genes. Conceptual figure created using Canva Pty Ltd. (https://www.canva.com/magic-design/, accessed on 26 August 2026).

Figure 1 summarizes the radiotherapy immune paradox and the proposed concept.

## 2. Review Methodology

This review is a structured, hypothesis-driven narrative synthesis rather than a systematic review or meta-analysis because the central question spans several partially independent fields: breast radiation-induced lymphopenia (RIL), immune dosimetry, nanoparticle radioenhancement, radiation immunogenicity, and fractionation/spatial/temporal dose modification. The objective was to determine whether these evidence streams can be integrated into a biologically coherent and experimentally testable framework. No PRISMA-style selection, formal risk-of-bias assessment, or meta-analysis was undertaken.

### 2.1. Literature Search and Evidence Domains

The evidence base was assembled through iterative, topic-targeted searches of PubMed/MEDLINE, Embase, Web of Science, and Scopus, together with citation tracking and retrieval of high-relevance articles. In total, 164 articles were retained, retrieved in full text, and individually reviewed, with no publication-date restriction. Studies were organized in a structured evidence map by disease/model, evidence setting, breast specificity, translational evidence level, nanoparticle or RT strategy, dose-exchange category, immune-dose-shaping dimension(s), principal tumor and immune endpoints, key contribution, and major limitation (Supplementary Table S1). The map served as an evidence inventory rather than a formal risk-of-bias or certainty assessment. Supplementary Table S1 represents the full evidence inventory identified and reviewed for this synthesis. The main-text reference list contains a curated subset selected for direct relevance to the specific claims developed in the manuscript, incremental evidentiary contribution, avoidance of redundant citation of concordant studies, and representative coverage across the predefined evidence domains. Inclusion in the evidence map, therefore, does not imply that every study was cited individually in the narrative synthesis. Additional details of the literature identification approach and representative search concepts are provided in Supplementary Methods S1.

The literature was grouped into five overlapping domains: breast RIL and immune dosimetry; nanoparticle-enhanced breast RT mechanisms; nanoparticle-enabled dose-exchange evidence; complementary immune-sparing strategies, including volume restriction, particle therapy, positioning, breath hold, fractionation, and FLASH; and indirect cross-tumor evidence used when breast-specific data were insufficient. Full-text assessment emphasized whether lower-dose NP + RT had been directly compared with higher-dose RT alone rather than whether dose reduction could merely be inferred from enhancement metrics.

### 2.2. Translational Evidence Hierarchy

Studies were interpreted using a four-level hierarchy of translational relevance rather than methodological quality. Level 1 comprised direct clinical breast-cancer evidence; Level 2, direct preclinical breast-cancer evidence; Level 3, indirect clinical or translational evidence from other malignancies; and Level 4, indirect preclinical, mechanistic, computational, methodological, review, or conceptual evidence. The hierarchy, therefore, reflects proximity to the clinical breast-cancer question rather than methodological rigor; indirect evidence was used primarily to define mechanisms, candidate endpoints, and testable hypotheses rather than breast-specific recommendations.

### 2.3. Classification of Nanoparticle Dose-Exchange Evidence

Because radiosensitization, radioenhancement, and dose reduction are used inconsistently across the nanoparticle literature [58,59,60,61], studies were classified according to the form of dose-exchange evidence provided. Category A denoted same-dose enhancement only. Category B denoted direct comparison of lower-dose NP + RT with higher-dose RT alone without a sufficiently rigorous tumor-isoeffect endpoint. Category C denoted formal dose–response or isoeffect evidence, including clonogenic dose–response curves, D50 or other isoeffect shifts, and sensitizer enhancement ratio/dose enhancement factor (SER/DEF) metrics derived from formal dose–response analysis [59,61,62,63]. Category D denoted claimed or implied dose reduction/low-dose efficacy without an appropriate higher-dose RT-alone comparator. Category E denoted insufficient information to classify. These categories describe evidence type rather than methodological quality; where experimental levels differed, in vitro and in vivo classifications were recorded separately.

### 2.4. Dual Interpretation of Tumor and Immune Outcomes

Tumor efficacy and immune preservation were not interpreted independently. Nanoparticle studies were evaluated for tumor-control and immunogenicity endpoints, whereas immune-sparing studies were evaluated for ALC, lymphocyte subsets, EDIC, distributed low-dose exposure, nodal/lymphoid and marrow exposure, fractionation, and functional outcomes. This reflects the principle that improvement on one side of the tumor–immune paradox does not establish improvement on the other. The review, therefore, distinguishes three non-equivalent quantities—physical radiation exposure, tumor biological effect, and immune biological effect—and treats the absence of their joint measurement as a central finding.

### 2.5. Cross-Tumor Extrapolation and Interpretation of Emerging Evidence

Evidence from other malignancies was included selectively when direct breast evidence was insufficient, including blood-irradiation modeling, marrow dose-volume effects, tumor-draining lymph-node biology, and FLASH-related immune effects [32,33,35,37,51]. Such evidence was not used to derive breast-specific dose constraints or justify omission of clinically indicated target volumes. Exploratory dosimetric thresholds, including EDIC, sternum dose, thymic dose, marrow V3–V20, and lung V1, were treated as hypothesis-generating pending independent validation.

To prevent the conflation of demonstrated evidence with translational extrapolation, Table 1 summarizes the principal claim boundaries underlying the proposed immune-dose-shaping framework.

## 3. The Two Immunological Consequences of Breast Radiotherapy

Radiotherapy exerts divergent effects on antitumor immunity: it can increase antigen availability and promote adaptive activation within the tumor while depleting radiosensitive lymphocytes across circulating, lymphoid, and hematopoietic compartments [1,39,64]. These effects depend on dose, fractionation, volume, geometry, baseline immune status, and tumor biology [6,24,47]. The relevant question is, therefore, not whether RT is immunostimulatory or immunosuppressive but which compartments are activated or injured, to what extent, and over what time scale.

### 3.1. Tumor Local Immunogenicity: From Radiation Damage to Adaptive Immune Activation

DNA damage can generate cytosolic double-stranded DNA and activate cGAS–STING/type I interferon signaling, while immunogenic cell death promotes calreticulin exposure and DAMP release, facilitating dendritic-cell maturation and CD8+ T-cell priming [1,3]. Breast-specific evidence supports several steps: cytoplasmic dsDNA and cGAS–STING signaling occur after photon and carbon-ion irradiation [3], and irradiated-tumor-cell products can increase effector-memory CD8+ responses that, combined with local RT and PD-L1 blockade, improve control of irradiated and nonirradiated tumors in a 4T1 model [4], although additional immune modulation was required for a robust response.

Radiation-induced immune activation is not monotonically dose-dependent. Excessive damage can trigger TREX1-mediated degradation of cytosolic DNA and attenuate cGAS–STING signaling [1], while dose per fraction and biologically effective dose can differentially regulate CD8+, NK-cell, and Treg responses in mammary tumor models [6]. In the randomized SIGNAL study, three-fraction neoadjuvant SBRT produced broader immune transcriptional and cellular changes than a single fraction [7], showing that larger dose per fraction cannot automatically be equated with greater immunogenicity. Sequence also matters: comparable cumulative exposures delivered in different high/low-dose orders produced different effector-to-regulatory balances [8]. Together, these findings support an immunogenic dose landscape shaped by fraction size, number, sequence, spatial distribution, and tumor context rather than a universal numerical “immunogenic dose” [65,66]. Partial-tumor irradiation generated STING-associated responses differing from full-tumor irradiation [46,47], while spatially fractionated irradiation produced distinct local and systemic antitumor immune effects in breast-tumor models [67], supporting spatial exposure as another variable for experimental exchange.

Human data complicate a purely activating interpretation. In the longitudinal PRADA/Neo-RT analysis, stromal tumor-infiltrating lymphocytes decreased significantly and had not recovered by surgery, with parallel peripheral-blood lymphodepletion [64], indicating that local RT effects can include depletion as well as activation. In 30 patients undergoing SBRT for bone metastasis, circulating PD-1 + CD8+ T cells were activated but so were Tregs, with greater Treg activation associated with poorer progression-free survival [68]. Immunogenic RT should, therefore, be evaluated using complementary molecular, cellular, and functional endpoints rather than a single favorable readout.

### 3.2. Systemic Immune-Cell Depletion: A Multicompartment Process

Only a small fraction of the total lymphocyte pool circulates at any time, with substantial populations residing in nodes, spleen, marrow, and tumor; clinically observed lymphopenia, therefore, likely integrates injury across dynamically connected compartments [32,33,39]. B cells appear particularly radiosensitive, T-cell subsets heterogeneous, and NK cells comparatively radioresistant, although intrinsic radiosensitivity alone does not fully explain treatment-related composition changes [39]. In 96 patients receiving adjuvant 3D-conformal RT, B-, T-, and NK-cell populations all fell significantly, regardless of prior chemotherapy [40]. Complementary longitudinal flow-cytometry data showed RT-associated shifts in circulating immune-cell composition, including increased CD4+ memory and regulatory T-cell subsets [69], supporting assessment of lymphocyte phenotype and function beyond total ALC.

Dosimetric evidence supports a graded exposure–response relationship. EDIC values corresponding to a 50% probability of grade ≥1, ≥2, and ≥3 lymphopenia were approximately 1.2, 2.1, and 3.7 Gy, respectively [22]. Distributed low-dose exposure also matters: full-DVH analysis identified volumes receiving as little as 1 Gy as predictors of lymphopenia, with ipsilateral-lung V1 among the most informative parameters [23]; earlier data linked greater lymphocyte decline to technique and broader low-dose lung exposure [70]; and interpretable machine learning likewise identified irradiated volume as an important determinant beyond prescription dose [71].

Hematopoietic and lymphoid structures add further exposure pathways. Sternum irradiation has been associated with clinically relevant lymphopenia using different dose-volume metrics [25,72], while thymic mean dose and low-dose exposure were incorporated into a predictive model for severe RIL [26]. Cross-tumor clinical evidence further supports a marrow contribution: in soft-tissue sarcoma, mean body and bone dose and low-dose bone-volume metrics, particularly bone V10 and V20, were associated with lower ALC nadir [35], while in chemotherapy-naïve patients receiving extended-field radiotherapy for prostate cancer, larger marrow V3–V10 predicted greater and persistent ALC decline, whereas a body-volume ≥ 2 Gy surrogate for circulating-blood exposure was not significantly associated [36]. Regional nodes are more complex because they are both potential disease sites and immune-priming structures; disease control remains the priority when nodal RT is indicated. Preclinical evidence shows that TdLN-derived effector CD8+ T cells migrate into irradiated tumors and contribute to repopulation after intratumoral depletion, with type I interferon signaling involved [37], reinforcing tumor–lymphatic–nodal–circulatory communication without justifying omission of indicated nodal irradiation. Systemic injury may also persist, with incomplete or prolonged lymphocyte recovery documented across cohorts [24,28,73,74], arguing against defining immune preservation from a single end-of-treatment count.

### 3.3. Why Tumor Immunogenicity and Lymphocyte Preservation Must Be Assessed Together

Neither intratumoral lymphocyte abundance nor peripheral ALC can independently represent radiation-induced immunity. RT may deplete and subsequently permit repopulation of intratumoral lymphocytes [37], activate tumor-reactive CD8+ T cells and Tregs simultaneously [6,68], and generate cGAS–STING activation without establishing functional systemic immunity. The relevant system is, therefore, better conceptualized as a tumor–lymphoid–blood–marrow network with potentially opposing effects across compartments [32,33,64,75]. PRADA illustrates this directly, with local and systemic immune depletion occurring within the same treatment pathway [64].

This distinction is particularly important for nanoparticle radioenhancement. Most nanoparticle studies assess tumor growth inhibition, radiosensitization, immunogenic cell death, or distant control without quantifying immune-compartment exposure, whereas the RIL/dosimetry literature measures depletion and physical exposure without establishing whether tumor immunogenicity is preserved. These studies address opposite sides of the same problem without testing their reconciliation. A successful immune-sparing strategy should, therefore, not be defined simply by a higher post-treatment lymphocyte count, any more than successful nanoparticle radioenhancement should be defined solely by greater tumor-cell killing. The goal is preservation of tumor control and immunogenic signaling together with reduced injury to the circulating, lymphoid, and hematopoietic compartments required to translate that signal into an effective antitumor response—the biological foundation for the framework developed below.

## 4. Clinical Significance of Radiation-Induced Lymphopenia in Breast Cancer

RIL is increasingly recognized as clinically relevant rather than a transient laboratory abnormality [39,40]. Its incidence varies with disease stage, chemotherapy, nodal irradiation, fractionation, and threshold definition, but contemporary cohorts consistently demonstrate measurable lymphocyte reduction [24,74,76]. Key uncertainties are how much depletion is attributable to radiation itself, which dosimetric features matter most, whether RIL is causally related to outcome, and whether it becomes especially relevant when subsequent treatment depends on intact antitumor immunity.

### 4.1. Incidence, Severity, and Kinetics

In a prospective multicenter pooled analysis of 607 patients receiving hypofractionated postmastectomy RT, mean nadir lymphocyte count was 0.75 ± 0.33 × 10^9^/L, 19.9% developed grade ≥3 RIL, and recovery to near-baseline required approximately one year [24]. Prior systemic therapy substantially influences magnitude: among 812 patients, grade ≥2 lymphocytopenia occurred in 19.4% without chemotherapy versus 45.1% with chemotherapy, with baseline lymphocytopenia and nodal irradiation important in both groups [76]. Pre-RT lymphopenia was present in 15.2% of 1012 patients, with prior chemotherapy the strongest determinant [77], indicating that nadir counts reflect both baseline vulnerability and additional radiation injury, and that studies ignoring pretreatment status may overattribute lymphopenia to radiation. In PRADA, the largest single decline occurred during the neoadjuvant RT interval itself (ALC 1.3→0.9 × 10^9^/L), although 93% received nodal irradiation [64].

Fractionation also influences kinetics. Comparing 50 Gy/25 fractions with 40.3 Gy/13 fractions after mastectomy, both produced substantial acute depletion, but six-month recovery favored the shorter schedule [78]. A post hoc analysis of 598 randomized postmastectomy patients likewise found hypofractionation associated with less RIL and higher nadir counts than conventional fractionation [29]. Persistent depletion occurs in selected populations: 26% of patients with residual nodal disease after neoadjuvant chemotherapy remained lymphopenic one year after RT [28], with other series similarly documenting sustained reductions [73,74,79]. These findings support longitudinal rather than single-timepoint assessment.

### 4.2. Dosimetric and Treatment Determinants

Whole-breast/chest-wall treatment, nodal irradiation, laterality, beam arrangement, intensity modulation, breath hold, particle therapy, and fractionation each alter blood, lung, heart, marrow, and lymphoid exposure differently; prescription dose alone, therefore, poorly represents relevant immune dose [43,45,80,81]. In 735 patients, modeled EDIC values associated with a 50% probability of grade ≥1/≥2/≥3 lymphopenia were approximately 1.2/2.1/3.7 Gy [22], although these remain exploratory research parameters. In 918 patients, full-DVH deep-learning analysis showed that volumes receiving even 1 Gy contributed to lymphopenia prediction, with ipsilateral-lung V1 among the most influential features [23]. Interpretable machine learning similarly suggested that irradiated volume may be more informative than incremental dose alone [71], indicating that reducing high-dose tumor exposure may be insufficient if broad low-dose distribution persists.

Treatment extent and technique are also important. Regional nodal irradiation is associated with greater depletion [31], and multi-region irradiation independently predicts lower nadir [24]. In a 163-patient locoregional breast-radiotherapy cohort, median EDIC was 4.23 Gy; right-sided treatment and regional nodal irradiation were associated with higher EDIC, with liver dose contributing to the laterality effect and integral dose to the nodal-volume effect [82]. Integrated photon-based RT was associated with lower nadir and slower recovery than a hybrid photon-electron approach [24]. Earlier data likewise linked RapidArc with greater decline and larger low-dose lung exposure [70], illustrating that improved high-dose conformity may coexist with increased low-dose volume [70,83].

Hematopoietic and lymphoid exposure adds further complexity. High mean sternum and heart doses were associated with grade 3 RIL, which occurred in 15.4% of a 156-patient cohort [72]; sternum D80 independently predicted clinically relevant lymphopenia in whole-breast RT without nodal treatment [25]; and thymic mean dose, V5, and V10, together with baseline ALC, technique, and internal mammary irradiation, predicted severe RIL with an AUC of 0.869 in 93 patients, 37.6% of whom developed grade ≥3 RIL [26]. These convergent but exploratory findings support a multicompartment organ-at-risk concept [32,34,39]; no single metric currently integrates baseline reserve, prior chemotherapy, treated volume, low-dose spread, nodal exposure, fraction number, technique, and hematopoietic/lymphoid exposure.

### 4.3. Prognostic Significance: Association Does Not Establish Causality

Associations between lymphopenia and inferior outcome are reproducible but do not establish causality [29,31,42,84,85,86,87,88]. Greater treatment-related lymphocyte reduction was associated with poorer disease-free survival in a randomized postmastectomy post hoc analysis [29]; greater RT-related decline predicted poorer recurrence-free survival after breast-conserving therapy [31]; persistent lymphopenia one year after RT predicted greater failure and death in residual node-positive disease [28]; and grade 3 RIL predicted substantially poorer DFS in a separate postmastectomy cohort [72].

More recent evidence introduces an important corrective. In 628 patients from a phase III randomized trial with 110.8-month median follow-up, only pretreatment peripheral lymphocyte count—not nadir count or recovery kinetics—remained independently associated with survival after multivariable adjustment [30]. In PRADA, baseline stromal TILs were not associated with relapse-free survival, whereas higher sTILs levels in residual tumor at surgery were [64], suggesting that post-treatment tissue immune composition may carry information distinct from peripheral counts. Takeda et al. likewise found lymphocytopenia prognostic only among chemotherapy-treated patients [76]. These findings suggest that part of the RIL–survival association may reflect baseline immune vulnerability rather than a causal effect of radiation-induced depletion. The most defensible conclusion is, therefore, that RIL is a reproducible prognostic marker without current proof of causality; the immune-dose-shaping hypothesis requires this relationship to be tested, not assumed.

### 4.4. RIL and Immunotherapy Efficacy: Indirect Evidence from Other Malignancies

Direct breast-specific evidence linking RIL to reduced immunotherapy efficacy remains insufficient. The strongest indirect evidence comes from stage III NSCLC, where severe lymphopenia at the start of consolidative immunotherapy after chemoradiation has repeatedly predicted inferior outcomes [89,90]. Modeled immune exposure adds further support: EDIC combined with baseline ALC stratified lymphopenia risk and treatment outcome after concurrent chemoradiation [91], while a pretreatment RIL-risk model showed durvalumab effectiveness varying by predicted lymphopenia risk [92].

These cross-tumor findings are mechanistically relevant given checkpoint inhibition’s growing role in TNBC, but there is currently no direct evidence that reducing EDIC, low-dose exposure, marrow dose, or lymphopenia during breast RT improves checkpoint-inhibitor efficacy. Breast prescriptions or target volumes should, therefore, not be modified on this basis. Instead, future breast radioimmunotherapy studies should prospectively assess immune preservation using baseline reserve, lymphocyte subsets, recovery kinetics, and tumor immune endpoints rather than ALC nadir alone. The central question is whether preventing avoidable immune injury improves therapeutic effectiveness, not simply whether lymphopenic patients fare worse. The principal breast-specific clinical evidence is summarized in Table 2.

## 5. Nanoparticle Platforms That Amplify Immunogenic Radiotherapy

Nanoparticle-mediated radioenhancement in breast cancer is mechanistically heterogeneous—some platforms increase energy deposition, while others alter redox/hypoxic state, interfere with DNA-repair, or deliver immune-active cargo—but share the property of increasing tumor biological response for a given physical exposure [5,9,10,11,12,15,16,18]. Most studies are designed around same-dose enhancement: greater killing, ROS, ICD, or STING signaling at the same dose establishes radioenhancement, not dose exchange, and the key question is whether this margin can instead reshape the exposure responsible for systemic immune injury [60,61].

### 5.1. High-Z Physical Radioenhancement

High-Z materials increase secondary-electron generation, concentrating energy deposition and increasing ROS, DNA double-strand breaks, and cell death [9,58,59]. Gold nanoparticles encapsulated in PEGylated liposomes produced a dose enhancement factor at 2 Gy of 2.56 ± 0.31 versus 1.79 ± 0.57 for commercial AuroVist in 4T1 cells, with increased ROS, DNA damage, and improved in vivo efficacy; however, the irradiated in vivo groups received the same physical dose, constituting Category A enhancement rather than dose exchange [9]. AS1411-functionalized gold nanoparticles also showed enhancement, with strong photon-energy dependence—approximately 1.33–1.34 at 100–190 kV versus 1.14 at 6 MV in MDA-MB-231 cells [52,53]—so kilovoltage effects cannot be assumed to persist under clinical megavoltage conditions.

Hafnium oxide NBTXR3 provides the most advanced clinical proof of concept. In the randomized Act.In.Sarc trial, intratumoral NBTXR3 with 50 Gy/25 fractions was compared with the same prescription without nanoparticles, demonstrating enhanced effect rather than dose reduction [55]. Mechanistically, NBTXR3 enhances DNA damage, micronucleus formation, and cGAS–STING signaling [13], with later evidence of immunopeptidome and T-cell receptor repertoire changes [14]. These non-breast data show that a physical radioenhancer can amplify immunologically relevant events beyond nonspecific killing. Clinical programs have thus far principally spent this margin on greater tumor response at the prescribed dose rather than exchanging it for a lower physical dose, a distinction central to the present hypothesis.

### 5.2. Redox and Tumor-Microenvironment Modulation

A second class amplifies radiation by modifying hypoxia, antioxidant capacity, or mitochondrial metabolism [10,17,93,94,95,96,97]. In 4T1 tumors, a folate-targeted ruthenium(II)/MnO_2_ liposomal construct (RMLF) used MnO_2_ to catalyze endogenous H_2_O_2_ and relieve hypoxia, Mn^2+^ to enhance cGAS–STING signaling, and ruthenium to promote mitochondrial dysfunction and mtDNA release [10]. The effect extended beyond radiosensitization to M1 macrophage polarization, dendritic-cell maturation, and increased memory T-cell populations after rechallenge, but all irradiated groups received the same dose, making this Category A evidence. Related manganese-, iron-, platinum-, or nanozyme-based platforms are attractive for immune-dose shaping because their amplification is tumor-context dependent [15,98,99], but this mechanistic complexity cannot be reduced to a single-dose-equivalent quantity without formal dose–response testing.

### 5.3. DNA-Damage and Repair Modulation

Nanoparticle-mediated interference with DNA-damage repair can shift radiation response and potentially reduce the dose required for a predefined effect [11,100]. The strongest breast-specific example is RAD50-siRNA nanoparticles in MDA-MB-231 cells: clonogenic assays across 2, 4, and 6 Gy shifted the dose for 50% clonogenic survival from approximately 5.5 Gy with RT alone to 2.0 Gy with NP + RT, an approximately 2.75-fold reduction in the dose associated with 50% clonogenic survival [11]. This is the clearest breast-specific demonstration of nanoparticle-enabled isoeffect dose exchange in the evidence set. However, the in vivo component used the same 10 Gy dose in both arms, so this study provides formal dose-exchange evidence in vitro but same-dose enhancement only in vivo, showing exactly where translational evidence currently stops.

PARP inhibition provides another route. A lutetium-based nanoplatform combined with olaparib in 4T1 tumors produced an SER of ~1.55 at 6 Gy, increased ROS, ICD signaling, cGAS–STING activity, dendritic-cell maturation, M1 polarization, and long-term immune memory after rechallenge [12]. The in vitro dose–response analysis provides Category C quantitative radiosensitization evidence, with SER derived from the 50% viability doses; in vivo, however, the same 6 Gy fractions were used across irradiated groups. The study, therefore, does not demonstrate nanoparticle-enabled in vivo dose exchange despite being framed as low-dose radioimmunotherapy [12].

### 5.4. STING Activation and Immune-Cargo Nanoplatforms

A fourth group engineers immune activation directly rather than solely as a downstream consequence of tumor killing [5,12,15,18,98,101,102,103]. Beyond RMLF-mediated endogenous STING amplification, R837/BMS@Au8 nanoparticles combining gold nanoclusters, TLR7 agonist R837, and PD-1/PD-L1 inhibitor BMS-1 enhanced dendritic-cell maturation and systemic responses at only 1 Gy in a bilateral 4T1 model [104]. Without a higher-dose comparator, however, this low nominal dose cannot be interpreted as a demonstrated dose reduction. Zeng et al.’s PARP/STING platform likewise combines radiosensitization with calreticulin exposure, p-TBK1/p-IRF3/IFN-β signaling, dendritic-cell maturation, and M1 polarization [12].

These platforms are especially relevant because they may preserve immunogenicity if physical exposure is subsequently reduced [5,12,15,18,98,105]. Existing studies, however, test whether NP + RT produces stronger immunity at the same or nominally low dose, not whether NP + reduced-exposure RT preserves tumor control and immunogenicity while reducing systemic immune injury, a sequence no breast study has yet demonstrated.

### 5.5. From Radiosensitization to Physical-Dose Exchange

An amplification margin has no immune-sparing consequence if the same dose, field, fractionation, and low-dose distribution are retained. Most in vivo breast nanoparticle studies [9,10,12,15,18,98,105,106,107,108], therefore, remain Category A, whereas several provide Category C evidence in vitro through formal dose–response, SER/DEF, or isoeffect analyses [5,11,19,52,53], which does not automatically mean clinically meaningful de-escalation. The clearest example is the D50 shift reported by Zetrini et al. [11] from approximately 5.5 to 2.0 Gy in vitro, contrasted with same-dose enhancement in vivo. This captures the current translational boundary: dose exchange is demonstrable at the cellular level, but in vivo breast evidence remains at the radiosensitization stage.

Cross-tumor evidence provides a complementary but non-substitutable precedent. In hypoxic head-and-neck cancer, modeled gold nanoparticle radiosensitization was translated into isoeffective exposures [54]. NBTXR3 at 2 Gy exceeded selected DNA-damage endpoints from 4 Gy RT alone, although this was a proximal damage endpoint rather than tumor control [13]. A multimodal glioblastoma platform combining gold nanoparticles, 5-ALA, and ultrasound at 2 Gy outperformed 10 Gy RT alone, but because multiple interventions were combined, the apparent 80% reduction cannot be attributed to nanoparticle enhancement specifically [109]. Clinical NBTXR3 studies demonstrate biological radioenhancement but not clinical dose exchange: Bonvalot et al. provide randomized same-dose evidence, whereas Le Tourneau et al. and Chajon et al. are noncomparative studies without RT-alone control arms [55,56,57].

Importantly, the amplification margin should not be considered transferable across nanoparticle classes. High-Z materials derive a substantial component of radioenhancement from physical energy-deposition effects and may, therefore, be strongly dependent on beam quality and radiation energy, whereas redox-, hypoxia-, DNA-repair-, metabolic-, or immune-modulating platforms rely more heavily on tumor biology, intracellular processing, pharmacokinetics, treatment timing, and microenvironmental state. Hybrid nanoplatforms may combine several of these mechanisms, further complicating attribution of the observed enhancement to a single pathway. Consequently, the magnitude, reproducibility, and most appropriate dimension of any putative dose exchange should be established separately for each nanoparticle–radiotherapy combination rather than extrapolated from one platform or mechanistic class to another.

Thus, nanoparticle-enabled dose exchange in breast cancer has been demonstrated most clearly in vitro and is supported by computational and cross-tumor precedents, but clean in vivo evidence that lower-dose NP + RT reproduces higher-dose RT-alone tumor control remains lacking. No breast study has further shown that such exchange simultaneously reduces systemic immune injury. The next experiment must, therefore, test whether a quantified nanoparticle-generated margin can be exchanged first for lower physical tumor dose and ultimately for reduced circulating, lymphoid, hematopoietic, geometric, or temporal exposure. The principal studies and strength of their dose-exchange evidence are summarized in Table 3.

## 6. Complementary Lymphocyte-Sparing Radiotherapy Levers

Nanoparticle radioenhancement is only one means of altering the tumor-effect/immune-injury balance. Breast RT can also be modified through geometry, volume, fractionation, particle selection, positioning, breath hold, and delivery time. These exposure components are not interchangeable, and several already have breast-specific evidence of reduced immune exposure [43,45,46,78]. Their relevance lies in combining a nanoparticle-generated amplification margin with such strategies rather than substituting for them; importantly, modality and fractionation may themselves modify the magnitude and biological character of that amplification margin.

### 6.1. Pharmacological Amplification Beyond Nanoparticle Radioenhancement

Any intervention that increases radiosensitivity or tumor immunogenicity could theoretically create an exchangeable margin, although evidence specifically targeting lymphocyte preservation remains limited. In a syngeneic 4T1 model, cabozantinib combined with hypofractionated RT modified CD8+, CD4+, Tregs, macrophages, and myeloid-derived suppressor cells across tumor, spleen, and TdLN [110], but without a lower-dose comparator, this does not establish pharmacological dose exchange. Nanoparticles may offer an advantage through tumor-preferential localization or local administration, whereas systemic radiosensitizers can also modify normal- or immune-tissue radiation response. The relevant quantity is, therefore, a favorable tumor-to-immune-compartment enhancement ratio, which should be measured rather than inferred from tumor efficacy alone.

### 6.2. Geometric Restriction, Particle Therapy, and Lymphoid Sparing

Geometric strategies can reduce immune exposure without changing prescribed tumor dose. In murine breast cancer, irradiation of approximately 40% of tumor area produced tumor control comparable with conventional irradiation while better preserving circulating and peritumoral lymphocytes and retaining dsDNA formation, DAMP release, and cGAS–STING activation [46]. This is among the closest existing studies to the proposed framework, although it did not involve nanoparticles. Partial-tumor irradiation likewise produced context-dependent inflammatory and STING responses distinct from full-volume exposure [47], supporting spatial exposure as an immunological variable without justifying omission of clinically indicated target tissue.

Clinical planning studies show that geometry and modality can substantially alter normal-tissue and low-dose exposure while preserving target coverage [111,112], while breast-specific EDIC studies demonstrate that treatment-design changes can also modify modeled immune exposure [43,44,45,82]. Lateral-decubitus positioning approximately halved EDIC and markedly reduced incidental level-I axillary dose compared with supine whole-breast irradiation when nodal RT was not indicated [45]. Intensity-modulated proton therapy reduced median EDIC from 3.37 to 2.13 Gy relative to VMAT, principally through lower integral dose [43], while proton-arc planning similarly reduced modeled EDIC in postmastectomy irradiation [112,113]. DIBH produced a smaller but measurable reduction in EDIC [44]. Together, these data show that target extent, incidental lymphoid exposure, integral dose, low-dose spread, positioning, and particle modality can be modified independently of tumor prescription [81,111].

These modality-dependent differences are relevant to nanoparticle-enabled immune-dose shaping for two distinct reasons. First, modalities that reduce integral dose, low-dose spread, or incidental lymphoid and marrow exposure may lower the immune cost of treatment independently of any nanoparticle effect. Second, the magnitude of nanoparticle radioenhancement itself may vary with beam quality and radiation energy. This is particularly important for high-Z platforms, for which secondary-electron production and resulting dose enhancement are energy-dependent; in breast-cancer models, enhancement observed at kilovoltage energies was substantially attenuated at 6 MV irradiation [52,53]. Consequently, a biological amplification margin quantified under one beam quality or modality should not be assumed to transfer unchanged to another. Different photon beam qualities, proton or electron therapy, brachytherapy, and ultra-high-dose-rate delivery may, therefore, require separate characterization of both nanoparticle enhancement and immune-relevant exposure before any exchange strategy is tested.

### 6.3. Fractionation and Repeated Blood-Pool Exposure

Repeated fractions create repeated opportunities for circulating lymphocytes to traverse irradiated fields. In postmastectomy breast RT, both conventional and hypofractionated schedules caused substantial acute lymphocyte depletion, but recovery favored the shorter regimen [78], while a larger randomized-trial analysis likewise associated hypofractionation with less RIL and higher nadir counts [29].

Dynamic blood-flow modeling provides a mechanistic explanation: in proton-treated hepatocellular carcinoma, reducing fractions from 15 to 5 increased predicted end-of-treatment ALC by approximately 17%, shortening beam-on time to 1 s by approximately 27.5% and combining both by approximately 60% [33]. These findings indicate that fraction number and delivery duration can independently influence circulating-blood exposure.

Shorter schedules, however, cannot automatically be considered immunologically superior. The SIGNAL study found broader immune changes after three fractions than one [7]; dose per fraction influenced CD8+-mediated activity and anti-PD-1 synergy in a mammary tumor model, with Treg responses modifying the effect [6]; and fraction sequence altered effector–regulatory balance [8]. Fraction number, dose per fraction, and sequence should, therefore, be treated as biologically distinct exchange variables.

Fractionation introduces an additional interaction with nanoparticle radioenhancement. Reducing the number of fractions may decrease repeated irradiation of recirculating lymphocytes and shorten cumulative treatment exposure, but changing dose per fraction can also alter tumor immunogenicity, lymphocyte injury, and the biological effect generated by the nanoparticle–radiation combination. These effects need not move in parallel; a schedule that reduces cumulative immune exposure may not necessarily preserve the same balance of cGAS–STING activation, effector-cell recruitment, regulatory-cell responses, and tumor control. Moreover, for nanoparticles whose activity depends on tumor retention, intracellular processing, redox modulation, or immune signaling, the interval between nanoparticle administration and irradiation and the spacing between fractions may influence the magnitude and persistence of radioenhancement. The relevant amplification margin should, therefore, be established under the same dose-per-fraction, fraction number, sequence, and treatment timing used in the subsequent immune-sparing experiment rather than extrapolated from a different fractionation regimen. Thus, modality and fractionation should be regarded not only as independent determinants of immune exposure but also as experimental context variables that may modify the magnitude and biological character of the nanoparticle-generated amplification margin.

### 6.4. Temporal Compression and FLASH Radiotherapy

FLASH extends immune-dose shaping into the temporal domain. Shortening delivery can reduce the blood volume traversing the irradiated field independently of any tissue-specific “FLASH effect.” In ex vivo human peripheral blood, ultra-high-dose-rate electrons produced fewer dicentric chromosomes than conventional dose rate, with lower BAX expression at 8 Gy [48]—evidence of reduced lymphocyte injury ex vivo, not clinical prevention of RIL.

Breast-specific evidence remains limited. In a syngeneic 4T1 model, FLASH maintained antitumor efficacy while reducing hepatic injury [49], whereas MV FLASH and conventional irradiation produced similar tumor control and tumor CD8+ infiltration but different splenic immune effects [114]. These findings reinforce that normal-tissue protection does not necessarily imply systemic immune preservation.

Modeling and cross-tumor experiments further caution against a simple “faster is better” principle. LymphoDose modeling showed that lymphoid-structure irradiation remains a substantial component of total lymphocyte-pool exposure even with ultra-high-dose-rate delivery [51]. Qin et al. found less circulating T-cell depletion but greater splenic depletion at higher dose rates [50], while dynamic blood-flow modeling indicates that high-dose-rate benefit principally reflects reduced circulating-blood exposure rather than direct sparing of resident lymphocytes in nodes, spleen, or marrow [33]. Temporal benefit, therefore, depends on dose rate, beam-on time, fractionation, and which immune compartments intersect the field.

### 6.5. From Independent Sparing Levers to Immune-Dose Shaping

Seven interacting dimensions of immune-relevant radiation exposure can be modified: physical dose, volume/spatial exposure, distributed low-dose exposure, marrow dose, geometry/modality, fractionation, and temporal/delivery exposure. Breast-specific studies show that spatial restriction can preserve lymphocytes and immunogenic signaling [46], geometry and modality can reduce modeled immune exposure [43,44,45,113], and fractionation can alter depletion and recovery [29,78]. Cross-tumor modeling further shows that delivery duration, recirculation, fractionation, and lymphoid dose cannot be reduced to prescription dose alone [33,51].

These seven dimensions should not be interpreted as independent, additive, or mathematically interchangeable quantities. Rather, they represent partially coupled components of radiation exposure that can influence different immune compartments through distinct mechanisms. Modification of one dimension may alter several others; for example, changing modality can simultaneously affect low-dose spread, integral dose, beam-on time, and marrow or lymphoid exposure, whereas changing fractionation alters both dose per fraction and cumulative recirculation of circulating lymphocytes through the treatment field. In this framework, ‘exchange’, therefore, denotes a testable redistribution of treatment exposure made possible by a quantified nanoparticle-generated amplification margin under a prespecified tumor-isoeffect constraint, rather than conservation of a single physical or biological quantity.

This framework is conceptual and hypothesis-generating: it organizes potentially modifiable exposure dimensions and defines testable relationships between nanoparticle-mediated tumor amplification and immune preservation, but it does not represent a clinically validated dose reduction strategy or establish treatment-planning constraints.

If nanoparticle enhancement permits lower tumor dose while fields, fractionation, and low-dose spread remain unchanged, systemic immune sparing may be limited. Conversely, the amplification margin could be allocated across several dimensions—lower physical dose, smaller volume where oncologically permissible, reduced integral or low-dose exposure, fewer fractions, altered geometry, or shorter delivery. Nanoparticle-enabled immune-dose shaping is, therefore, broader than dose de-escalation: the objective is to redistribute a quantified biological amplification margin across selected exposure dimensions while preserving tumor control and immunogenicity and reducing injury to circulating, lymphoid, and hematopoietic compartments.

**Figure 2:**
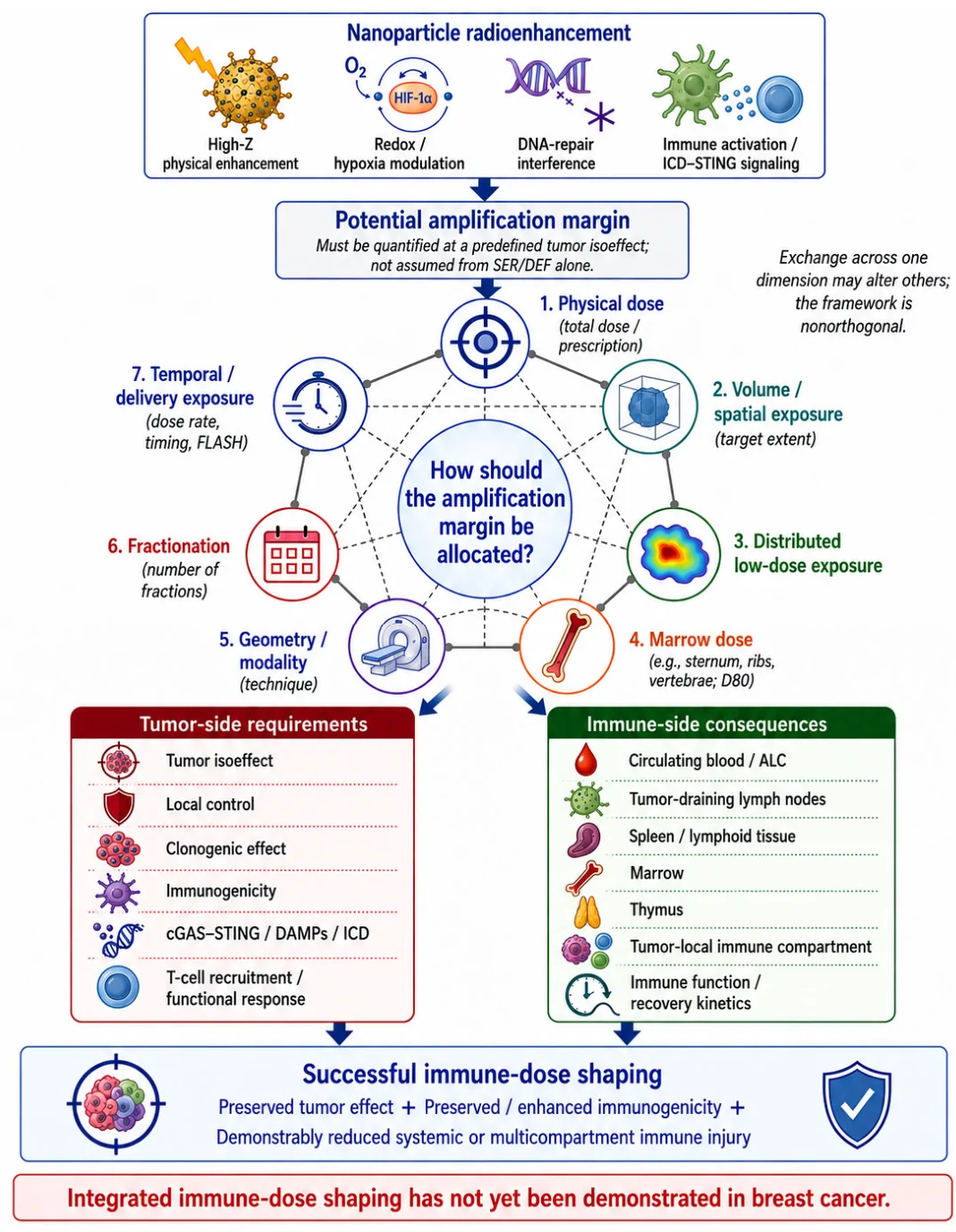
**Seven-dimensional framework for nanoparticle-enabled immune-dose shaping in breast cancer radiotherapy.** Nanoparticle radioenhancement may create a biological amplification margin that can potentially be redistributed across seven interacting, nonorthogonal exposure dimensions: physical dose, volume/spatial exposure, distributed low-dose exposure, marrow dose, geometry/modality, fractionation, and temporal/delivery exposure. The dimensions are partially coupled rather than independent; modifying one may alter several others simultaneously, and no fixed conversion factor between dimensions is implied. These dimensions may differentially affect circulating blood, lymphoid organs, marrow, tumor-associated immune cells, and tumor response. Successful immune-dose shaping would require preserved tumor isoeffect and immunogenicity together with demonstrably reduced systemic or multicompartment immune injury. This framework is hypothesis-generating and has not yet been validated clinically. Conceptual figure created using Canva Pty Ltd. (https://www.canva.com/magic-design/, accessed on 26 August 2026).

The key question is not simply whether nanoparticles permit less RT but which component of exposure should be exchanged to achieve the greatest immune preservation without sacrificing tumor effect or immunogenicity. Complementary strategies relevant to this question are summarized in Table 4, and the seven interacting and nonorthogonal exchange dimensions are shown in Figure 2.

## 7. Can Tumor Immunogenicity and Lymphocyte Preservation Be Jointly Optimized?

Tumor immunogenicity and systemic immune preservation should not be treated as inevitably opposing objectives: the central optimization problem is to identify an exposure pattern providing adequate tumor local immunogenic effect while minimizing avoidable immune-system injury. If nanoparticles increase tumor biological effect, the resulting margin can be spent conventionally (maintaining prescription, increasing tumor damage) or exchanged for modified dose, volume, fractionation, or delivery while preserving a predefined tumor effect, the second use underlying immune-dose shaping, contingent on three questions: whether a reproducible immunogenic dose window exists; whether nanoparticle enhancement can maintain treatment within that window at lower physical immune exposure; and whether physical dose, tumor effect, and immune effect can be measured separately rather than assumed to move in parallel.

### 7.1. The Immunogenic Dose Window Is a Landscape Rather than a Single Dose

The dose–immunogenicity relationship is not monotonic: TREX1 induction at high single-fraction doses can attenuate cGAS–STING signaling in select systems [1], and in the AT3-OVA model, dose per fraction rather than BED determined local CD8+ engagement and anti-PD-1 synergy, while NK-cell-dependent control depended more on BED, and high-dose-per-fraction schedules generated stronger Treg accumulation, demonstrating simultaneous activation of antitumor and immunoregulatory pathways [6]. Clinical data reinforce this: three-fraction SIGNAL schedules produced broader immune changes than single fraction [7], and reversing high/low-dose fraction order substantially alters effector-regulatory balance despite similar cumulative exposure [8]. Spatial exposure adds another dimension—partial-tumor irradiation produces distinct STING responses from full-tumor irradiation, and reduced-area irradiation preserves tumor control, dsDNA formation, DAMPs release, cGAS–STING activation, and circulating/peritumoral lymphocytes simultaneously [46,47]. The useful concept is, therefore, an immunogenic dose landscape rather than a fixed threshold, with different immune endpoints potentially having different optima. Reducing physical exposure does not necessarily move treatment away from this optimum and could, in principle, reduce excessive local immune suppression while decreasing systemic injury; indiscriminate reduction, however, could fall below the exposure required for adequate cytotoxicity or innate signaling. The objective is the lowest immune-cost exposure that preserves a predefined tumor-control and immunogenic effect, not simply the lowest dose.

### 7.2. From a Tumor Dose Multiplier to an Immune-Dose-Shaping Tool

Most nanoparticle studies implicitly treat the platform as a tumor dose multiplier, holding prescription constant while maximizing damage. Zetrini et al. provided the clearest breast-specific evidence that a quantifiable in vitro amplification margin can be exchanged, although this remains unconfirmed in vivo [11]. The critical next step is deciding what to exchange for that margin. Reducing nominal tumor dose alone may produce considerably less immune sparing than anticipated if low-dose volume, nodal exposure, and beam geometry remain unchanged, given the predictive importance of low-dose volumes such as V1 in breast RIL models [23]. Alternatively, the margin could support volume exchange, building on Chen et al.’s 2024’s non-nanoparticle proof of concept [46], or fractionation exchange, though local consequences would need independent verification given that changing dose per fraction or sequence can alter immunity in ways not predicted by total dose [6,7,8], or temporal/delivery exchange, since shortening beam-on time can reduce circulating-pool exposure with benefit depending on patient-specific lymphocyte kinetics [33]. An enhancement factor cannot be arithmetically converted into an equivalent clinical dose reduction—radiosensitization depends on energy, concentration, distribution, oxygenation, and endpoint, so any exchange must be demonstrated empirically for the relevant tumor endpoint, preferably in vivo [58,59,61].

### 7.3. Physical Dose, Tumor Biological Effect, and Immune Effect Are Distinct Quantities

These three quantities are frequently conflated but are not interchangeable. Physical dose describes deposited energy and its distribution (DVHs, integral dose, low-dose volumes, fractionation, beam-on time, dynamic blood-exposure estimates). Tumor biological effect describes what that exposure does to malignant tissue (clonogenic survival, growth delay, DNA damage, ICD, cGAS–STING, local control), the level at which nanoparticle radioenhancement primarily acts. Immune effect is a third, more distributed dimension (lymphocyte depletion/preservation, subset and functional state, lymphoid/hematopoietic injury, tumor and nodal immune composition, distant control, memory) that cannot be reliably inferred from either physical dose or tumor response alone. A geometrically smaller field can preserve peritumoral lymphocytes while retaining cGAS–STING activation without changing dose per irradiated region [46]; conversely, circulating lymphocytes and stromal TILs both declined during PRADA treatment, yet baseline sTILs retained predictive value for pathologic complete response despite this treatment-related depletion [64]—immune-cell abundance changes should not be equated with tumor immunogenicity. Similarly, SER/DEF/D50 metrics quantify tumor radiosensitivity but not systemic sparing: Zetrini et al. [11] provided strong tumor-level dose-exchange evidence but none that the lower in vitro isoeffective dose translates into less lymphocyte injury in vivo.

Success, therefore, requires the preservation of tumor effect together with a demonstrated—not assumed—favorable immune effect across relevant circulating, lymphoid, hematopoietic, and tumor local compartments. These requirements are operationalized as the five-component proof standard in Section 9.6. The goal is no longer to maximize the NP + RT-versus-RT-alone difference but to test whether nanoparticle radioenhancement can decouple tumor biological effect from systemic immune exposure—unproven in breast cancer but experimentally tractable given convergent existing evidence.

## 8. Breast Cancer Subtype and Clinical Setting Considerations

The rationale for immune-dose shaping is unlikely to be uniform across breast cancer; subtype, baseline infiltration, systemic therapy, extent of nodal irradiation, and treatment intent all influence its potential value, and this framework is not a universal argument for de-escalation but identifies contexts where the balance is particularly testable.

### 8.1. Triple-Negative Breast Cancer as the Most Immediate Testing Context

TNBC more frequently shows substantial lymphocytic infiltration with direct clinical relevance to antitumor immunity. CD8+ tumor infiltration carried prognostic significance in ER-negative but not ER-positive disease in a randomized cohort [41], and higher EDIC was independently associated with poorer event-free survival specifically in TNBC [42]—convergent tissue- and dosimetry-level evidence supporting TNBC as a priority testing context. Several evidence strands converge here: the disproportionate representation of TNBC models such as MDA-MB-231 and 4T1 in the breast nanoparticle-radiosensitization literature [21]; the clearest measurable breast-specific in vitro dose-exchange evidence [11]; and direct dose/fractionation–immunity investigation in mammary models, including the dose-per-fraction/BED dissociation reported by Sia et al. [6] and the human neoadjuvant schedule comparison reported by Spears et al. [7]. TNBC should be viewed as a priority experimental context rather than the exclusive beneficiary, since neither preferential clinical benefit nor enhanced immunotherapy response has been established. The rationale is strongest because immune-mediated biology is comparatively visible and measurable here.

### 8.2. Hormone Receptor-Positive and HER2-Positive Disease

The weaker prognostic contribution of CD8+ infiltration in ER-positive disease [41] suggests local immune activation may be a less dominant determinant, providing less direct rationale for introducing a nanoparticle specifically for immune preservation. However, RIL severity depends on geometry, volume, baseline reserve, and nodal irradiation regardless of subtype [22,24,71,76], so immune-dose reduction may retain value even where local activation is less prominent, shifting the objective toward minimizing avoidable systemic injury while preserving established local control. HER2-positive disease involves complex sequential cytotoxic/targeted/immune-modulating exposures for which current evidence is insufficient for subtype-specific recommendations; subtype should be prospectively recorded rather than treated as background.

### 8.3. Neoadjuvant Radiotherapy as a Mechanistically Informative Setting

Because tumor tissue remains available after irradiation, this setting permits direct peripheral-intratumoral comparison linked to pathological response [115]. The PRADA analysis illustrates this: circulating lymphocytes declined during chemotherapy and further during RT, with stromal TILs also decreasing [64]; paired blood/tissue measurement reveals dynamics invisible in postoperative-only designs, providing a practical setting for testing whether a reduced-exposure regimen preserves tumor response alongside ALC, TILs, and innate-signaling endpoints. Its value in this framework is primarily mechanistic rather than as a general standard-care platform.

### 8.4. Adjuvant Whole-Breast and Regional Nodal Radiotherapy

Here, tumor tissue is unavailable, but systemic exposure can be studied under standardized conditions, making this setting suitable for validating dosimetric/hematologic components. Whole-breast irradiation without nodal treatment offers relative control for relating positioning, breath-hold, particle therapy, and fractionation to immune-cell exposure, with planning studies demonstrating substantial EDIC modification without omitting indicated targets [43,44,45,113]. Regional nodal irradiation is more consequential: larger volumes and nodal treatment are consistent RIL determinants [31], and near-universal nodal irradiation accompanied substantial ALC decline in PRADA [64], findings that argue not for omitting indicated nodal irradiation but for reducing unnecessary exposure around required targets, distinguishing intentional treatment from incidental irradiation of adjacent structures. Immune-dose shaping must operate within oncologic target requirements, optimizing integral dose, low-dose spread, beam arrangement, fractionation, and delivery time without providing biological justification for compromising established coverage.

### 8.5. The Immunotherapy Context

Systemic lymphocyte preservation may matter most when RT is intended to cooperate with immunotherapy, since checkpoint inhibition requires functional immune populations, though direct breast evidence that reducing RIL improves immunotherapy efficacy is currently lacking—the argument remains inferential, supported by cross-tumor data [89,90,91,92]. Nanoparticle-enhanced ICD, STING signaling, and immune-cell recruitment do not establish that lowering systemic exposure will enhance checkpoint efficacy; the appropriate next step is measuring local immunogenicity, systemic preservation, and therapeutic response concurrently, particularly in TNBC where the engagement rationale is strongest. A treatment causing substantial immune depletion alongside increased inflammatory signaling may generate an incomplete response, just as a lymphocyte-sparing regimen weakening antigen release or innate sensing may preserve cells without adequate stimulus.

### 8.6. Prioritizing Clinical Settings for Translation

The available evidence prioritizes immune-responsive models, particularly TNBC, for initial experimental testing; neoadjuvant studies for tissue-based mechanistic validation; and adjuvant cohorts for multicompartment dosimetry and peripheral immune assessment. Subtype and setting do not change the central hypothesis but determine where it can be tested most convincingly. The sequence for translating these settings into progressively more clinical testing is developed in Section 10.

## 9. Proposed Dual-Endpoint Research Framework

The central hypothesis can only be tested by designs measuring tumor efficacy and systemic immune preservation within the same experiment. The essential comparison is not simply NP + RT versus RT alone but whether a nanoparticle-enhanced regimen delivered with less physical immune exposure can preserve tumor control and immunogenicity while reducing injury to circulating, lymphoid, and hematopoietic compartments.

### 9.1. Experimental Comparison Arms

A minimum design requires six groups: sham control; nanoparticle alone; standard RT; NP + standard RT; reduced-exposure RT alone; and NP + reduced-exposure RT. The NP + standard-dose arm confirms genuine radioenhancement; the reduced-exposure RT-alone arm rules out reduced radiation exposure alone as the explanation for preserved response. The nanoparticle-alone arm is equally necessary: many radioenhancers are not immunologically inert—platforms activating STING, redox biology, or carrying immune cargo can alter lymphocyte viability, trafficking, or cytokine signaling independent of irradiation [15,16,98]—so without this arm, improved immune preservation after NP + reduced-exposure RT cannot be attributed specifically to reduced radiation exposure rather than direct nanoparticle pharmacology. The reduced exposure should derive from prior dose–response experiments identifying an isoeffective dose [11], extended in vivo via dose-escalation/de-escalation series using durable tumor-growth delay, local control, and, where appropriate, survival rather than short-term volume alone. A parallel volume-exchange series should compare full-field RT with NP-enhanced restricted-volume RT, building on the non-nanoparticle proof of principle [46]. Dose and volume exchange should be studied separately before combining with fractionation or delivery-time modification, since combining variables in one experiment would make it impossible to attribute the source of any observed benefit.

### 9.2. Tumor-Control and Immunogenicity Endpoints

Tumor isoeffect should be established using clonogenic dose–response curves in vitro and durable tumor-growth delay or local-control endpoints in vivo, with survival incorporated where appropriate. Preserved tumor control alone is insufficient; the regimen must retain immunogenic effects across cytosolic dsDNA, cGAS–STING, type I interferon, DAMPs release, dendritic-cell activation, CD8+/Treg/NK composition, and, where feasible, abscopal response and memory. The choice of immune endpoint must account for fractionation, since dose per fraction and BED can differentially regulate CD8+, NK-cell, and Treg responses [6]—equal tumor control after two schedules cannot be assumed to imply equal immunogenicity. Success requires NP + reduced-exposure RT to achieve a prespecified equivalence margin relative to standard RT while preserving or enhancing immunogenic endpoints, with that margin defined before analysis rather than inferred from nonsignificant differences in small groups.

### 9.3. Systemic Immune Preservation

Peripheral ALC alone is insufficient because it obscures redistribution among subsets and cannot indicate functional competence, and circulating blood represents only a fraction of the total lymphocyte pool. Studies should distinguish cell number, phenotype, localization, and function, assessing serial subsets, activation/exhaustion/memory phenotypes, and functional assays before, during, at nadir, and through recovery, sampling TdLNs, spleen, thymus, and marrow in parallel. Human studies should extend beyond single post-RT measurements to longitudinal ALC, nadir, recovery kinetics, and functional assays where feasible, as illustrated by the value of PRADA’s serial paired measurements [64]; clinical outcome associations such as EDIC’s link to event-free survival in TNBC [42] support relevance without establishing causality.

### 9.4. Multicompartment Dosimetry

Quantifying “less immune exposure” requires multiple physical layers. Circulating-blood exposure is as follows: static EDIC is a useful approximation associated with lymphopenia severity in breast RT [22], but dynamic models treating circulating cells as an organ at risk across fractions are preferable for mechanistic testing [32], since fraction number and beam-on time can substantially alter predicted nadir independent of treatment intent [33]; blood-dose assessment could include mean blood dose, low-dose blood-volume metrics such as V0.1/V0.5/V1, and cumulative fractional exposure. Lymphoid-organ exposure (TdLNs, spleen, thymus) should distinguish intentional target dose from reducible incidental exposure rather than treating nodes as simple avoidance structures. Hematopoietic exposure should include the sternum and, where relevant to the irradiated field, other marrow-bearing structures; breast-specific associations between sternum dose and lymphopenia provide direct support for this component [25,72]. Temporal exposure (fractions, beam-on time, sequence, dose rate) matters because very short delivery may reduce circulating-blood irradiation without ensuring protection of resident lymphocytes in lymphoid or hematopoietic compartments; multicompartment modeling and preclinical dose-rate data demonstrate that benefit in circulating blood need not parallel effects in spleen or other tissues [50,51]. Emerging spatiotemporal blood-dose frameworks move beyond single EDIC estimates toward this integration [34,51], though no single model yet captures all components sufficiently for routine use.

### 9.5. Nanoparticle Biodistribution

The immune benefit of reduced radiation exposure will depend partly on whether the radioenhancer is preferentially localized to the tumor rather than immune compartments. Nanoparticle biodistribution can involve liver, spleen, marrow, and lymphoid tissues depending on platform and route of administration, and local nanoparticle concentration may modify the biological radiation effect beyond that predicted from physical dose alone [58]. Studies should quantify concentration in tumor, blood, spleen, nodes, marrow, and liver at the time of irradiation, ideally summarized, as proposed here, by a tumor-to-immune-compartment radioenhancer ratio, addressing whether the nanoparticle preferentially amplifies radiation’s biological effect in a tumor versus immune tissue. The nanoparticle-alone arm described in Section 9.1 is what makes distinguishing direct immunomodulation from reduced-exposure sparing experimentally possible.

### 9.6. Defining Experimental Success

**Figure 3:**
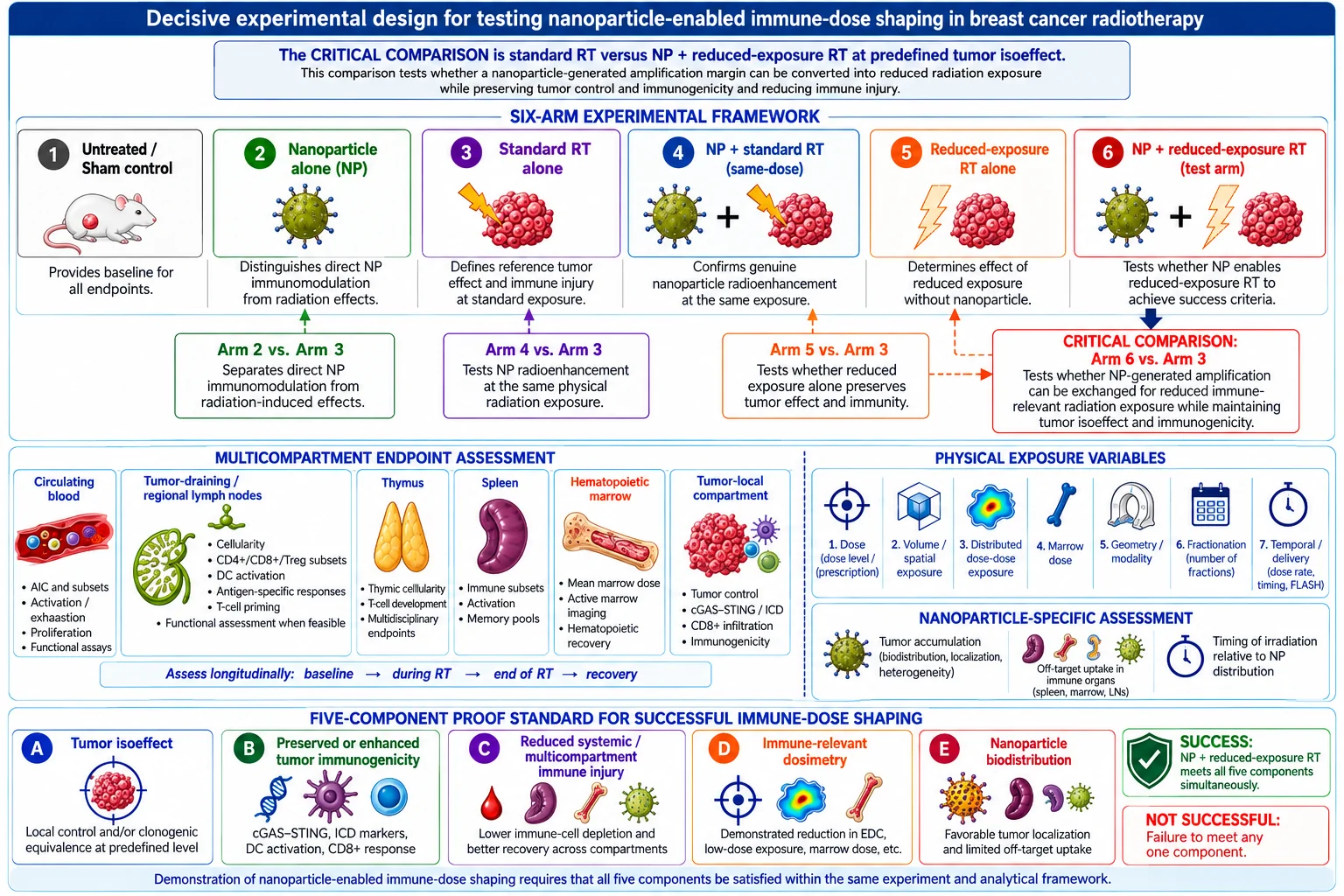
**Decisive experimental design for testing nanoparticle-enabled immune-dose shaping in breast cancer radiotherapy.** The six-arm framework separates nanoparticle effects, radiation effects, same-dose radioenhancement, reduced-exposure effects, and the combined strategy. The critical comparison is **standard RT versus NP + reduced-exposure RT** at predefined tumor isoeffect. The nanoparticle-alone arm distinguishes direct nanoparticle immunomodulation from radiation-related immune preservation, the NP + standard RT arm confirms genuine radioenhancement, and the reduced-exposure RT-alone arm determines whether lower exposure alone explains the observed effect. Demonstration requires the same five-component proof standard as Table 5, assessed within one experimental framework: (**A**) tumor isoeffect; (**B**) preserved/enhanced immunogenicity; (**C**) reduced multicompartment immune injury; (**D**) immune-relevant dosimetry documenting the intended exposure reduction; and (**E**) nanoparticle biodistribution confirming favorable tumor-to-immune-compartment localization. Reproducible radioenhancement and a quantifiable margin are prerequisites for testing this standard. The framework is evidence-informed and has not yet been validated clinically. **Abbreviations:** ALC, absolute lymphocyte count; DC, dendritic cell; EDIC, effective dose to immune cells; ICD, immunogenic cell death; LN, lymph node; NP, nanoparticle; RT, radiotherapy; Treg, regulatory T cell; cGAS–STING, cyclic GMP–AMP synthase–stimulator of interferon genes. Conceptual figure created using Canva Pty Ltd. (https://www.canva.com/magic-design/, accessed on 26 August 2026).

As a conceptual experimental framework intended to guide proof-of-concept studies rather than clinical implementation, we propose that nanoparticle-enabled immune-dose shaping should be considered demonstrated only when five components are assessed within the same experimental framework: (1) tumor isoeffect, with tumor control maintained within a predefined equivalence range relative to standard RT; (2) preserved or enhanced tumor immunogenicity, rather than inferred from tumor response alone; (3) demonstrably reduced systemic or multicompartment immune injury, including circulating, lymphoid, and hematopoietic compartments; (4) immune-relevant physical or dynamic dosimetry, documenting the intended reduction in one or more exposure dimensions; and (5) nanoparticle biodistribution, confirming tumor-preferential or otherwise favorable localization relative to immune compartments. Reproducible tumor radioenhancement and a modified regimen that exploits the resulting amplification margin are prerequisites for testing this five-component proof standard rather than separate success endpoints. The most convincing result would show NP + reduced-exposure RT is tumor-isoeffective to standard RT, preserves or enhances local immunogenic signaling, and produces less injury to circulating, lymphoid, and hematopoietic compartments, integrating tumor-level exchange [11], spatial/fractionation-immunity evidence [6,46], and clinical EDIC–lymphopenia and outcome evidence [22,42]. Equally informative would be negative results: preserved control with weakened immunogenicity means the exchange is oncologically adequate but immunologically incomplete; preserved immunogenicity without reduced injury means immune-dose shaping has not been achieved; improved preservation with inferior tumor control is unacceptable regardless of magnitude. The reframed question is as follows: can the biological gain created by the nanoparticle be redistributed to reduce immune exposure while maintaining tumor control and immunogenicity? The decisive six-arm experimental design and five-component proof standard proposed for testing this hypothesis are summarized in Table 5 and Figure 3.

## 10. Staged Translational Development Pathway

Translation requires a staged sequence in which each step resolves a different uncertainty, with failure at an earlier stage preventing escalation. The stages outlined below are proposed research development steps rather than clinically validated criteria for nanoparticle-enabled radiation de-escalation.

**Stage I: Quantifying the Amplification Margin.** Formal dose–response experiments must precede immune-sparing studies. The D50 shift from 5.5 → 2.0 Gy is the clearest breast-specific in vitro dose-exchange example [11], but the immediate gap is independent replication of this isoeffect shift in additional models and in vivo confirmation, not another same-dose enhancement experiment. Progression beyond Stage I requires a reproducible, quantitatively defined in vivo margin demonstrated across independent experiments and, where feasible, more than one biologically relevant model, with prespecified uncertainty around the isoeffect estimate.

**Stage II: Controlled Preclinical Immune-Dose-Shaping Studies.** Once quantified, the margin should be tested in the six-arm design described in Section 9.1, modifying a single dimension at a time—physical dose first, then irradiated volume separately, before introducing fraction-number or delivery-time changes—since dose, volume, fractionation, and temporal compression can each modify immunity through different mechanisms. Volume and fractionation changes affect tumor and immune outcomes through distinct pathways [6,46], so an apparently favorable result from changing several variables simultaneously would be mechanistically uninterpretable. Multicompartment sampling (blood, tumor, nodes, spleen, thymus, marrow) and nanoparticle biodistribution should be measured longitudinally alongside radiation-delivery modeling. The strongest result would be an NP + reduced-exposure RT regimen tumor-isoeffective to standard RT, preserving or improving local immunogenic signaling, and reducing systemic injury across more than one compartment.

**Stage III: Biomarker-Rich Early Clinical Translation.** Human translation should emphasize pharmacodynamic and feasibility endpoints over efficacy. The neoadjuvant setting offers particular advantages because tumor tissue remains accessible, permitting simultaneous ALC kinetics, subset, TILs, and mechanistic-signaling assessment, as paired PRADA measurements illustrate [64], despite not evaluating mechanistic immunogenicity endpoints. A biomarker-rich study should first establish safety, tumor deposition, and pharmacokinetics with standard-of-care RT exposure before introducing a predefined reduced-exposure cohort; dose reduction should not be the first clinical question for an untested platform. Adjuvant breast RT provides a complementary standardized setting combining serial hematologic measurement with EDIC and marrow/lymphoid dosimetry, supported by demonstrated exposure–lymphopenia relationships [22,23,25] and EDIC’s link to event-free survival in TNBC [42], though these associations do not establish causal benefit from lowering EDIC. Stage III should be considered successful only if feasibility, safety, tumor delivery, reduced immune exposure, and preservation of the intended tumor-biological effect can all be demonstrated in the same clinical setting.

**Stage IV: Prospective Comparative Testing.** Only after biological feasibility is demonstrated should the strategy enter comparative testing, no longer justified by radiosensitization alone. An initial randomized comparison of standard RT with NP + reduced-exposure RT could use a co-primary or hierarchical endpoint incorporating treatment-plan immune exposure and longitudinal preservation alongside predefined oncologic non-inferiority criteria, with the biological isoeffect evidence established during stages I–II informing regimen selection, while the clinical non-inferiority margin should be prespecified independently using clinically justified criteria. TNBC may represent the most informative initial context, though selection should also consider baseline lymphocyte count, systemic treatment, nodal irradiation, and nanoparticle biodistribution. Trials combining this strategy with checkpoint inhibition represent a later rather than initial step, since direct evidence that reducing lymphopenia improves immunotherapy outcomes in breast cancer is currently absent.

**Stage V: Personalizing the Exchange.** If feasible, the eventual goal is not a single universal prescription. Tumor-side variables (uptake, oxygenation, radiosensitivity, subtype) and immune-side variables (baseline ALC, radiosensitivity, recovery kinetics, marrow reserve) both influence the amplification margin and immune cost. Modeled benefit from altered fractionation/beam-on time has been shown to vary with individual lymphocyte radiosensitivity and recovery kinetics [33]. The eventual question becomes not “how much can dose be reduced” but “how should the margin be allocated in this patient to achieve the greatest reduction in immune cost while preserving tumor effect?”—closer to personalized optimization than uniform de-escalation.

**Figure 4:**
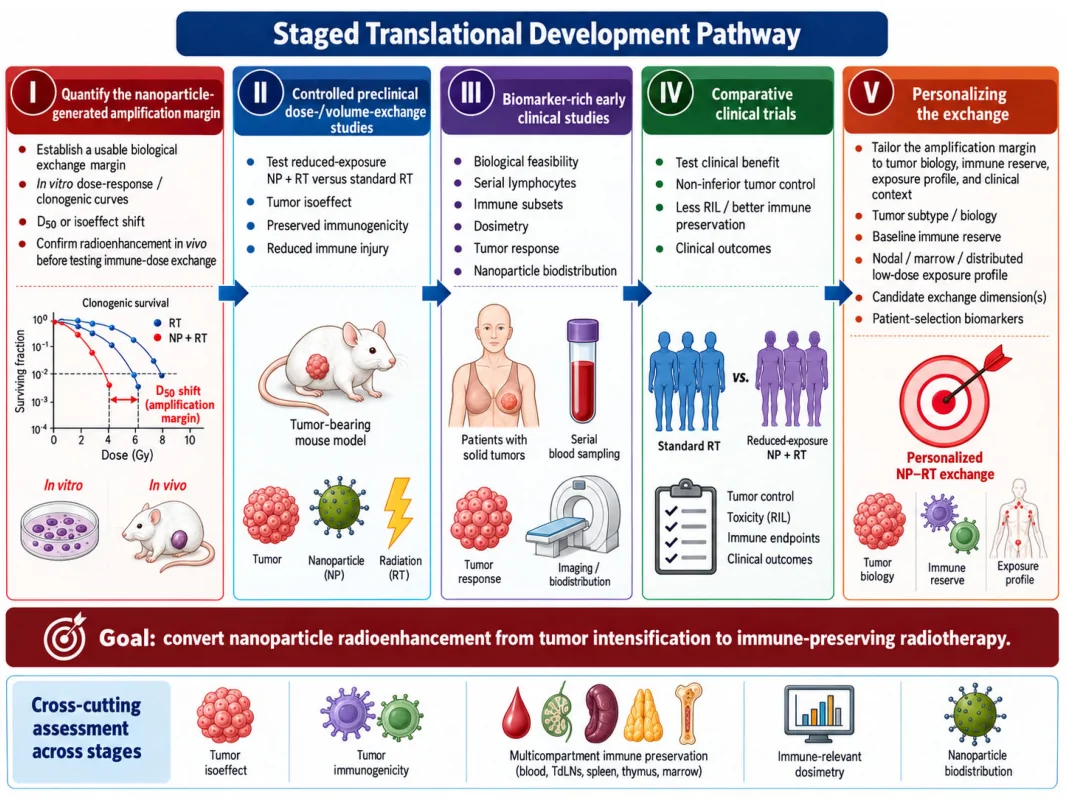
**Staged translational development pathway for nanoparticle-enabled immune-dose shaping.** Translation progresses from quantitative definition and in vivo confirmation of the amplification margin, through controlled preclinical exchange and biomarker-rich early clinical investigation, to prospective comparative testing and ultimately patient-specific allocation of the margin. Progression between stages requires preservation of tumor isoeffect and immunogenicity together with evidence of multicompartment immune preservation, relevant dosimetry, nanoparticle biodistribution, and safety. NP, nanoparticle; RIL, radiation-induced lymphopenia; RT, radiotherapy; TdLN, tumor-draining lymph node. Conceptual figure created using Canva Pty Ltd. (https://www.canva.com/magic-design/, accessed on 26 August 2026).

**Progression Criteria.** These progression criteria are proposed here as an evidence-informed framework, not an established regulatory standard. A candidate platform should not proceed from radiobiological characterization to immune-dose-shaping experiments without a quantified margin; should not proceed to human reduced-exposure studies without in vivo tumor isoeffect and measurable immune preservation; should not progress to comparative testing without nanoparticle safety and favorable tumor localization relative to immune compartments; and clinical claims of immune benefit should require direct biological evidence rather than inference from a lower prescription alone. This sequence—quantify the margin, demonstrate preclinical exchange, establish dual tumor–immune benefit, validate in humans, then test comparative efficacy—converts the hypothesis into discrete, falsifiable questions. The resulting staged translational pathway—from quantitative amplification margin definition to personalized exchange—is summarized in Figure 4.

## 11. Safety and Translational Barriers

Nanoparticle-enabled immune-dose shaping introduces two distinct risks: those of the nanoparticle itself and those created by deliberate modification of radiation exposure. These should be evaluated separately before jointly, because a platform may be an effective radioenhancer yet unsuitable for immune-dose shaping because of unfavorable biodistribution, direct immune toxicity, heterogeneous uptake, or insufficiently reproducible enhancement.

**Biodistribution and Immune-Compartment Exposure.** Systemically administered nanoparticles may accumulate in liver, spleen, marrow, and lymphoid tissues according to size, charge, coating, administration route, and clearance kinetics [58]. For high-Z platforms, local secondary-electron generation could increase the biological radiation effect in these compartments, so a reduced nominal RT dose cannot itself establish immune sparing. Biodistribution should, therefore, be quantified at the time of irradiation across tumor and relevant immune compartments and, where possible, expressed as the tumor-to-immune-compartment radioenhancer ratio proposed in Section 9.5. Intratumoral NBTXR3 demonstrates the feasibility of localized delivery [55] but not systemic immune preservation; intravenously administered platforms require independent compartment-specific assessment.

**Direct Nanoparticle Immune Effects.** Many radioenhancers directly influence oxidative stress, STING signaling, macrophage polarization, or checkpoint biology [5,15,16,98], complicating causal attribution. Increased CD8+ infiltration or preserved ALC after NP + reduced-exposure RT could reflect direct nanoparticle immunomodulation, reduced radiation injury, or both; the nanoparticle-alone arm proposed in Section 9.1 is intended to distinguish these effects. Functional assessment should extend beyond routine hematology because unchanged cell counts may coexist with altered activation, exhaustion, or antigen presentation, whereas transient peripheral reductions may reflect redistribution rather than injury.

**Heterogeneity and Reproducibility.** Heterogeneous nanoparticle uptake is more consequential when radioenhancement is used to justify reduced radiation exposure than when it supplements an unchanged prescription. Poorly penetrated tumor regions may otherwise receive neither adequate amplification nor the original radiation effect. Future studies should, therefore, quantify spatial nanoparticle distribution relative to viable tumor rather than total concentration alone, with imaging-based surrogates validated against biological effect. The in vitro D50 shift reported by Zetrini et al. [11] should not be assumed reproducible across tumors, irradiation conditions, or nanoparticle concentrations without independent confirmation.

**Radiation Energy and Platform Transferability.** High-Z radioenhancement is strongly energy-dependent: in one breast-cancer study, dose enhancement of approximately 1.33–1.34 at 100–190 kV fell to approximately 1.14 at 6 MV [52]. An enhancement observed under orthovoltage conditions should, therefore, not be transferred directly to clinical megavoltage planning, while proton, electron, brachytherapy, and FLASH delivery may involve different determinants. Non-high-Z platforms depend more strongly on tumor biology and delivery timing. Radioenhancement should, therefore, be regarded as platform-, context-, and endpoint-specific rather than as a universal multiplicative factor.

**Oncologic Safety of Exchange.** The evidentiary threshold must be higher when an amplification margin is used to reduce radiation exposure than when it is used to increase effect at unchanged dose. Breast-specific formal dose-exchange evidence remains strongest in vitro, whereas an independent in vivo demonstration of tumor isoeffect between lower-exposure NP + RT and higher-exposure RT alone is still lacking. Restricted-volume studies provide mechanistic support [46,47] but do not justify omission of clinically indicated target tissue; specifically, any theoretical immune advantage from reduced incidental lymphoid dose must not be conflated with withholding indicated regional nodal irradiation.

**Manufacturing and Regulatory Reproducibility.** Once a nanoparticle is used to justify reduced radiation exposure, batch-to-batch variation in size, charge, loading, and stability become clinically consequential. Physicochemical specifications should, therefore, be linked to retained radioenhancing activity, potentially through companion uptake or functional enhancement assays. The nanoparticle and radiation regimen should ultimately be evaluated as an interdependent therapeutic strategy rather than as independent components.

**Avoiding False Equivalence.** Lower prescription dose is not synonymous with lower immune toxicity. A reduced tumor dose may yield limited systemic benefit if the same blood volume, low-dose spread, fraction number, or lymphoid/marrow exposure persists. Conversely, modest prescription reduction combined with substantial decreases in distributed low-dose or multicompartment exposure could produce greater immune benefit. The relevant endpoint is, therefore, a demonstrable reduction in biological immune-system exposure and injury; lower physical dose or improved EDIC remains an intermediate mechanistic endpoint rather than proof of preserved immune function.

**Translational Boundary.** This translational boundary is proposed here as an evidence-informed safety framework, not an established regulatory standard. Clinical NBTXR3 experience shows that meaningful nanoparticle-mediated biological amplification is achievable in patients [55], while breast preclinical studies establish radiosensitization, in vitro dose-exchange evidence, and immune activation, and clinical breast studies show that immune exposure and RIL are measurable and modifiable. The translational boundary defined in Section 9.6 should, therefore, be maintained: progression beyond proof of concept requires satisfaction of the proposed five-component proof standard rather than improvement in any single dosimetric, tumor, or immune endpoint. Until such integrated evidence is available, nanoparticle-enabled immune-dose shaping should remain a research strategy rather than a rationale for clinical de-escalation.

## 12. Evidence Gaps and Prioritized Research Agenda

The literature is sufficiently developed to support a testable hypothesis but not clinical implementation. The principal weakness is fragmentation across domains rather than absence of data; the priority is, therefore, to integrate tumor, immune, dosimetric, and nanoparticle measurements within the same experimental framework.

**Independent Replication of Dose Exchange.** Breast-specific formal dose-exchange evidence remains predominantly in vitro, with the clearest example provided by Zetrini et al. [11]; a clean in vivo comparison of lower-dose NP + RT with higher-dose RT alone has not been identified. Future studies should generate complete dose–response curves and test candidate platforms across more than one biologically relevant model differing in radiosensitivity, immune phenotype, and hypoxic burden.

**Direct Demonstration of Immune Benefit.** No study has yet shown that a nanoparticle-enabled reduction in physical exposure reduces systemic immune injury while preserving tumor isoeffect. This is the decisive experiment underlying the hypothesis, and the six-arm design proposed in Section 9.1 provides one approach for separating radioenhancement, direct nanoparticle immunomodulation, and reduced-exposure sparing.

**Defining Tumor and Immunogenic Isoeffect.** Tumor-control equivalence does not guarantee immune equivalence. Sia et al. [6] provided one of the clearest breast-specific demonstrations that fractionation can produce divergent immune consequences despite comparable antitumor effects, but this dissociation has not been independently replicated in a second breast model, itself a research gap rather than merely supporting evidence. Future studies should, therefore, define tumor-control and immunogenic-quality equivalence separately and map multidimensional immunogenic dose landscapes rather than assume a universal numerical target.

**Functional Multicompartment Immunology.** ALC alone cannot distinguish depletion from redistribution or numerical preservation from functional impairment. Future studies should, therefore, incorporate phenotype, localization, and function, with longitudinal sampling of circulating blood and relevant lymphoid and hematopoietic compartments, including TdLN, spleen, thymus, and marrow, since redistribution can mimic depletion while numerical preservation can coexist with functional impairment.

**Integrated and Nanoparticle-Aware Immune Dosimetry.** Static EDIC [22] and low-dose-volume metrics such as V1 [23] should be complemented by dynamic fraction-by-fraction models [32,33], with lymphoid and marrow exposure quantified separately. Multicompartment approaches such as the BLOOD framework and related models [34,51] are important methodological advances but are not yet validated breast-specific standards. Because local nanoparticle concentration can modify biological effect at the same physical dose, biodistribution should also be incorporated into immune-dose estimates, including the tumor-to-immune-compartment radioenhancer ratio proposed in Section 9.5.

**Identifying the Optimal Exchange Dimension.** The relative immune-sparing value of reducing physical dose, irradiated volume, distributed low-dose exposure, marrow dose, geometric spread, fraction number, or delivery time remains unknown. Factorial designs should identify which dimension—or combination—provides the greatest immune benefit per unit of preserved tumor effect. Nodal and lymphoid exposure should be mapped quantitatively where relevant, without implying omission of clinically indicated regional treatment.

**Defining the Optimal Population and Treatment Context.** TNBC provides a strong initial rationale but may not represent the population deriving the greatest eventual benefit. Effect modification by subtype, baseline ALC, systemic therapy, treatment geometry, nanoparticle biodistribution, and immune reserve should be studied prospectively. The association between EDIC and event-free survival in TNBC [42] remains clinically relevant but does not establish that lowering EDIC improves outcomes.

**Integration With Immunotherapy.** Whether immune preservation enhances the therapeutic value of RT-generated tumor immunogenicity remains unresolved and should be tested only after nanoparticle-enabled exchange and immune preservation are established. Later trials should incorporate immune-relevant dosimetry, longitudinal immune profiling, and tumor biomarkers rather than treating RT exposure as binary.

**Reporting Standards.** Future studies should report nanoparticle physicochemical characteristics, administration timing, quantitative biodistribution, radiation parameters, formal tumor-isoeffect methodology, and longitudinal functional immune endpoints. Same-dose radioenhancement, quantitative radiosensitization, formal dose exchange, and demonstrated immune-dose shaping should remain explicitly distinguished.

**Prioritized Sequence.** This sequence mirrors the staged translational pathway in Section 10: first reproduce nanoparticle-enabled dose exchange quantitatively in vivo and pair it with direct measurement of immune preservation; only then add spatial, fractionation, or temporal complexity, followed by refinement of dynamic dosimetry, biodistribution, and functional immune assays. Clinical translation and immunotherapy integration should follow only after these mechanistic requirements are satisfied. The central question is no longer whether nanoparticles can enhance breast RT but whether that amplification can be converted into a measurable reduction in immune cost without sacrificing tumor control or immunogenicity.

## 13. Limitations

This is a hypothesis-driven translational review rather than a systematic review or meta-analysis. The literature search was iterative and topic-targeted across PubMed/MEDLINE, Embase, Web of Science, Scopus, and citation tracking and was not designed to guarantee exhaustive capture; no PRISMA selection process, formal risk-of-bias assessment, or quantitative evidence grading was performed. The evidence base combines clinical breast cohorts, preclinical models, cross-tumor translational studies, computational dosimetry, and nanoparticle radiobiology that differ substantially in biological context, radiation energy, fractionation, and clinical relevance. The four-level evidence hierarchy makes this heterogeneity explicit but cannot eliminate uncertainty from extrapolation across models and disease sites.

The nanoparticle literature remains dominated by same-dose radioenhancement rather than formal dose-exchange designs, and breast-specific evidence supports the biological plausibility of exchange more strongly than its in vivo or translational reproducibility. The proposed immune-dose-shaping framework is, therefore, assembled from complementary studies rather than validated within a single integrated experiment. In addition, current immune dosimetry incompletely captures patient-specific blood flow, lymphoid and marrow exposure, temporal effects, and nanoparticle distribution, so neither reduced physical dose nor improved modeled immune exposure can yet be assumed to represent biological immune preservation.

Associations between RIL or modeled immune exposure and clinical outcome are predominantly observational and remain susceptible to confounding by disease burden, systemic therapy, and baseline immune reserve; whether immune preservation causally improves outcomes requires prospective testing. Likewise, the proposed translational pathway, progression criteria, success criteria, and safety boundaries are evidence-informed, author-derived constructs rather than established field or regulatory standards and are intended to generate falsifiable experimental questions rather than clinical guidance.

Finally, the framework emphasizes breast cancer, particularly TNBC, because nanoparticle-radioenhancement evidence is concentrated in TNBC models. Generalizability to hormone receptor-positive or HER2-positive disease may, therefore, be limited by differences in tumor immune context and systemic therapy. Conversely, several mechanistic and dosimetric principles derive partly from other malignancies and may not transfer quantitatively to breast radiotherapy. These limitations reinforce the central distinction that nanoparticle-enabled immune-dose shaping is a testable translational hypothesis, not an established clinical strategy.

## 14. Conclusions

Breast radiotherapy creates a fundamental immunological tension: radiation can promote DNA damage, immunogenic cell death, cGAS–STING activation, and adaptive antitumor immunity within the tumor, while exposure of circulating blood, lymphoid tissue, and marrow can produce radiation-induced lymphopenia and weaken the systemic immune resources required to sustain those effects. Nanoparticle radioenhancement may help reconcile these opposing consequences by creating a biological amplification margin that could be exchanged, at least in part, for reductions in immune-relevant radiation exposure. This concept extends beyond physical-dose reduction to volume/spatial exposure, distributed low-dose exposure, marrow dose, geometry/modality, fractionation, and temporal delivery, provided oncologic target requirements are preserved.

The available evidence supports biological plausibility rather than clinical implementation. Breast studies demonstrate that immune exposure is measurable and treatment-dependent, while nanoparticle studies establish substantial radioenhancement and limited formal dose-exchange evidence. The critical next step is, therefore, integrated validation of the proposed framework in vivo before any clinical reduction in radiation exposure is considered.

The central question is, therefore, no longer simply whether nanoparticles can make radiotherapy more effective but whether the biological gain they generate can be redistributed to make radiotherapy more selective in immunological terms, preserving or enhancing tumor local immune effects while reducing avoidable systemic immune injury. If demonstrated experimentally, nanoparticle-enabled immune-dose shaping could represent a distinct translational strategy for reconciling radiotherapy-induced tumor immunogenicity with systemic immune preservation in breast cancer.

## Figures and Tables

**Table 1:** Claim boundaries for nanoparticle-enabled immune-dose shaping in breast cancer radiotherapy.

Evidence Status	What Can Currently Be Claimed	What Cannot yet Be Claimed
**Demonstrated**	Breast RT can cause radiation-induced lymphopenia; immune exposure is influenced by treatment volume, low-dose exposure, marrow/lymphoid irradiation, geometry/modality, fractionation, and delivery characteristics. Nanoparticles can produce measurable tumor radioenhancement and, in selected models, increase immunogenic signaling.	These observations do not show that nanoparticle use reduces systemic immune injury.
**Supported but incomplete**	Quantitative nanoparticle-mediated dose exchange is biologically plausible and has been demonstrated in a breast-specific in vitro isoeffect analysis [11]; several non-NP breast studies also show that spatial or fractionation changes can alter immune exposure or immune response.	In vivo breast tumor isoeffect between lower-exposure NP + RT and standard RT has not been independently established.
**Plausible and testable**	A nanoparticle-generated amplification margin could potentially be redistributed across seven interacting dimensions—physical dose, volume/spatial exposure, distributed low-dose exposure, marrow dose, geometry/modality, fractionation, and temporal/delivery exposure—to reduce immune cost while preserving tumor effect.	The optimal exchange dimension, magnitude of exchange, and patient population are unknown.
**Not demonstrated**	—	No reviewed study has simultaneously shown tumor isoeffect + preserved/enhanced immunogenicity + reduced systemic/multicompartment immune injury, with immune-relevant dosimetry and nanoparticle biodistribution measured within the same nanoparticle-enabled reduced-exposure experiment.

Evidence-status categories are descriptive, not a formal certainty-of-evidence grading system. “Demonstrated” indicates direct evidence for that component only, not validation of immune-dose shaping as an integrated strategy. “Supported but incomplete” denotes evidence for part of the framework without full in vivo or translational confirmation. “Plausible and testable” identifies hypotheses supported by convergent but indirect evidence. “Not demonstrated” denotes absence of an integrated experiment satisfying the proposed dual tumor–immune proof standard.

**Table 2:** Clinical breast-cancer evidence linking radiotherapy exposure, lymphopenia, immune-compartment dose, and outcome.

Study	Population and RT Setting	Immune/Exposure Endpoint	Key Dosimetric or Treatment Determinant	Main Finding	Relevance to Immune-Dose Shaping
Chen et al., 2022 [22]	735 patients with breast cancer receiving adjuvant RT	Peripheral lymphocyte count; RIL; EDIC	Estimated dose to circulating immune cells	Lymphocyte counts decreased in 92.7% of patients and 60.5% developed post-RT lymphopenia. Increasing EDIC was associated with greater lymphocyte depletion; exploratory EDIC values associated with a 50% probability of grade ≥1, ≥2, and ≥3 RIL were approximately 1.2, 2.1, and 3.7 Gy, respectively.	Core quantitative breast-specific evidence linking physical immune exposure with RIL. EDIC thresholds should be regarded as exploratory associations rather than validated planning constraints.
Yu et al., 2022 [71]	Breast cancer patients receiving adjuvant RT; interpretable machine-learning analysis	RIL/ALC reduction	Treatment volume and multiple clinical/dosimetric variables	Identified treatment-related and patient-specific determinants of RIL and demonstrated that lymphopenia risk reflects multiple interacting exposure variables rather than prescription dose alone.	Supports a multivariable rather than single-organ interpretation of immune injury and provides an early bridge between conventional dosimetry and later full-DVH analyses.
Yoon et al., 2023 [31]	531 patients after breast-conserving surgery and RT without chemotherapy	ALC reduction; clinically meaningful RIL; recurrence-free survival	Regional nodal irradiation	Regional nodal irradiation was a major treatment-related determinant of lymphocyte depletion; greater ALC reduction was also associated with poorer recurrence-free survival.	Demonstrates the potential immune cost of expanding treated lymphoid volume, while not implying omission of oncologically indicated nodal RT.
Kobzeva et al., 2023 [40]	Patients with primary breast cancer receiving RT	Peripheral lymphocyte subsets	RT exposure	RT altered the composition of circulating lymphocyte populations rather than affecting only total ALC.	Supports moving beyond total lymphocyte counts toward phenotype-specific immune assessment.
Chen et al., 2024 [23]	918 patients with stage I–III breast cancer; multicenter development and external validation cohorts	Post-RT lymphopenia predicted using full DVH information and deep learning	Very-low-dose exposure, particularly ipsilateral-lung V1	DVH variables across a wide dose range contributed to lymphopenia prediction; exposure at doses as low as 1 Gy was informative, with ipsilateral-lung V1 particularly influential.	Strong evidence that distributed low-dose exposure matters, supporting immune-dose shaping beyond prescription/high-dose metrics alone.
Yoneyama et al., 2024 [64]	PRADA cohort: 30 high-risk breast cancer patients receiving systemic therapy followed by preoperative locoregional RT; longitudinal tumor and blood sampling	ALC and stromal TILs dynamics	Neoadjuvant locoregional RT; 93% received nodal irradiation	Median ALC fell from 1.9 × 10^9^/L at baseline to 1.3 after systemic therapy, 0.9 after NART, and 0.8 before surgery; the largest additional decline occurred during RT. sTILs also declined during treatment.	Rare human paired systemic–tumor immune dataset. Demonstrates that RT-associated immune effects occur simultaneously in blood and tumor tissue, although immune-cell depletion should not be equated with loss of tumor immunogenicity.
Zhao et al., 2025 [24]	607 patients from 8 institutions receiving predominantly hypofractionated postmastectomy RT	Serial PLC; RIL severity and recovery	Multi-region targets, internal mammary irradiation, integrated photon techniques	RIL occurred in 81.5% and grade ≥3 RIL in approximately 20%. Greater target extent and integrated photon techniques were associated with lower nadir PLC; recovery extended for months.	Provides large multicenter evidence linking treatment extent and technique with both acute RIL severity and prolonged recovery.
Loap et al., 2025 [82]	163 patients with locally advanced breast cancer receiving locoregional helical tomotherapy	EDIC	Laterality, regional nodal irradiation, liver dose, integral total dose	Median EDIC was 4.23 Gy. Right-sided treatment and regional nodal irradiation increased EDIC; liver dose contributed to the laterality effect and integral dose to the nodal-volume effect.	Direct breast-specific evidence identifying spatial and treatment determinants of modeled systemic immune exposure.
Ni et al., 2025 [72]	156 patients receiving postmastectomy RT	RIL severity; disease-free survival	Heart dose, sternal dose, integrated RT, BMI	Grade 3 RIL occurred in 15.4%. Severe lymphopenia was associated with markedly poorer DFS; high mean heart and sternum doses, integrated RT, and low BMI were associated with grade 3 RIL.	Connects dosimetric determinants, severe RIL, and clinical outcome within one cohort, strengthening the case that immune exposure may be clinically relevant rather than merely hematological.
Sun et al., 2026 [25]	112 patients receiving conventional whole-breast RT after breast-conserving surgery; nodal fields excluded	Grade ≥2 lymphopenia; serial PLC	Sternal marrow dose, particularly D80	Grade ≥2 lymphopenia occurred in 57.1%. Sternum D80 remained independently associated with RIL; an exploratory threshold near 2.85 Gy was reported.	Direct breast-specific evidence that the hematopoietic compartment contributes to RIL, extending the framework beyond circulating blood.
Xia et al., 2025 [26]	93 consecutive patients receiving adjuvant breast RT	Grade ≥3 RIL	Thymic mean dose, V5 and V10; RT technique; IMNI	Grade ≥3 RIL occurred in 37.6%. Thymic mean dose, V5, V10, baseline ALC, RT technique, and internal mammary nodal irradiation were associated with severe RIL; a combined model achieved AUC 0.869.	Adds the thymus as a clinically measurable lymphoid compartment, supporting multicompartment immune dosimetry. Reported cutoffs require prospective validation.
Loap et al., 2026 [42]	TNBC treated with helical tomotherapy	EDIC; event-free survival	Effective dose to immune cells	Higher EDIC was associated with poorer event-free survival in TNBC.	Particularly important because it extends EDIC beyond prediction of lymphopenia to a breast-subtype-specific clinical outcome association, while remaining observational and non-causal.
Cheptea et al., 2025 [45]	Breast cancer patients receiving whole-breast irradiation without nodal indication; paired treatment-planning comparison	EDIC and incidental axillary lymphatic dose	Patient positioning	Ipsilateral lateral-decubitus positioning reduced estimated systemic immune exposure and incidental level-I axillary irradiation compared with supine planning.	Provides breast-specific human planning evidence that clinically permissible changes in setup can materially alter both systemic and regional lymphoid exposure without changing the indication for whole-breast RT.
Loap et al., 2024 [43]	Locoregional breast RT planning in 10 patients	EDIC and integral dose	IMPT versus VMAT	Median EDIC decreased from approximately 3.37 Gy with VMAT to 2.13 Gy with IMPT; reduction in integral dose was the main contributor.	Establishes in a breast-specific clinical planning setting that radiation modality can substantially modify estimated immune dose, supporting EDIC as a treatment-sensitive rather than purely patient-specific quantity.
Loap et al., 2025 [44]	Right-sided locoregional adjuvant breast RT	EDIC	DIBH and fractionation	DIBH reduced EDIC in both fractionation schedules; hypofractionated plans produced lower EDIC than conventionally fractionated plans. For example, median EDIC with DIBH was approximately 2.15 Gy with hypofractionation versus 2.81 Gy with conventional fractionation.	Provides breast-specific evidence that estimated immune exposure is sensitive to more than one treatment-design variable, supporting consideration of geometry and treatment duration together.
Yuan et al., 2018 [78]	Patients receiving postmastectomy RT with conventional versus hypofractionated schedules	Serial peripheral lymphocyte counts and recovery	Fractionation schedule	Both regimens produced substantial acute lymphocyte depletion, whereas subsequent lymphocyte recovery favored the shorter treatment schedule.	Provides direct clinical breast evidence that fractionation is associated with different lymphocyte kinetics, complementing dosimetric studies that estimate rather than directly measure immune effects.

Studies were selected to represent principal breast-specific evidence linking radiotherapy with lymphocyte depletion, immune-compartment exposure, modifiable treatment determinants, and outcome; the table is not exhaustive. Planning studies without measured lymphocyte endpoints were included when they showed the physical feasibility of modifying estimated immune exposure. Reported EDIC, marrow, thymic, and other dose thresholds are exploratory, study-specific associations, not validated planning constraints. Associations between RIL or modeled immune exposure and outcome do not establish causality or that reducing exposure improves survival or efficacy; likewise, reducing a surrogate such as EDIC does not itself establish biological immune preservation. The evidence therefore supports the measurability and potential modifiability of immune-relevant exposure, not validated thresholds or de-escalation recommendations.

**Table 3:** Nanoparticle radioenhancement and strength of evidence for physical-dose exchange.

Study	Tumor Model/Setting	Nanoparticle Strategy	RT Conditions	Quantitative Radioenhancement	Dose-Exchange Evidence	Immune Effects	Interpretation
Zetrini et al., 2024 [11]	MDA-MB-231 TNBC in vitro; breast-cancer xenograft in vivo	RAD50-silencing siRNA nanoparticles targeting DNA double-strand-break repair	In vitro 2–6 Gy; in vivo 10 Gy	Clonogenic D50 decreased from approximately 5.5 Gy with RT alone to 2.0 Gy with NP + RT	C—formal dose–response/isoeffect evidence in vitro; in vivo same-dose enhancement only	Primarily DNA-damage/radiosensitization study rather than immune focused	Strongest direct breast-specific evidence for a measurable NP-generated dose-exchange margin. The exchange is demonstrated in vitro, not in vivo.
Huynh et al., 2021 [54]	Hypoxic head-and-neck cancer; cross-tumor computational model	Gold nanoparticles	Fractionated RT modeling, including 2 Gy/fraction	Predicted dose for tumor control decreased from 69.9 ± 5.8 Gy without AuNP to 57.2 ± 5.1 Gy with weekly and 53.0 ± 4.0 Gy with biweekly AuNP administration	C—formal modeled isoeffect/dose exchange	Not assessed	Cross-tumor computational proof of principle. Demonstrates mathematically how radiosensitization could be exchanged for lower physical dose, but is neither experimental nor breast-specific and depends on model/SER assumptions.
Marill et al., 2019 [13]	CT26.WT colorectal model; cross-tumor preclinical evidence	NBTXR3 hafnium oxide nanoparticles	2 and 4 Gy comparisons	NBTXR3 + 2 Gy produced greater DSB/micronucleus effects than RT alone at 4 Gy	B—direct lower-dose NP + RT versus higher-dose RT-alone comparison, by proximal DNA-damage endpoint only	cGAS–STING/immune-related mechanisms investigated within the broader NBTXR3 program	Important experimental dose-exchange precedent, but not breast-specific and not demonstrated at the level of tumor control or survival.
Chiang et al., 2021 [109]	Orthotopic glioblastoma; cross-tumor in vivo precedent	GoldenDisk AuNP platform combined with 5-ALA and ultrasound	Multimodal treatment with 2 Gy versus 10 Gy RT alone	2 Gy multimodal treatment produced superior tumor effects and survival compared with 10 Gy RT alone	B—lower-dose NP-containing multimodal treatment versus higher-dose RT alone	Antitumor mechanistic effects reported; not primarily lymphocyte focused	In vivo physical-dose reduction precedent, but the effect cannot be attributed specifically to the nanoparticle because the intervention was multimodal.
Choi et al., 2025 [9]	4T1 breast cancer	Au-loaded liposomes	In vitro 0–6 Gy; in vivo 6 Gy	DEF at 2 Gy approximately 2.56; comparator Au formulation approximately 1.79	C in vitro—quantitative dose–response/DEF evidence; A in vivo—same-dose enhancement	Not a major immune-focus study	Strong quantitative breast radiosensitization, but no direct demonstration that a lower NP-enhanced dose can replace a higher RT-alone dose in vivo.
Tudda et al., 2022 [52]	MDA-MB-231 breast cancer in vitro	15-nm gold nanoparticles	100 kV, 190 kV, and 6 MV; multiple radiation doses	DEF approximately 1.34 at 100 kV, 1.33 at 190 kV, and 1.14 at 6 MV	C—quantitative dose–response radiosensitization; no direct lower-dose-versus-higher-dose isoeffect experiment	Not assessed	Demonstrates strong radiation-energy dependence and cautions against extrapolating kilovoltage enhancement to clinical megavoltage breast RT.
Zeng et al., 2026 [12]	4T1 TNBC in vitro and in vivo	Organolutetium-based nanoparticle plus olaparib	6 Gy in vivo	SER approximately 1.55	C in vitro—formal dose–response/SER evidence; A in vivo—same-dose 6 Gy comparison	Enhanced cGAS–STING signaling, ICD, DC maturation, M1 polarization, CD4+/CD8+ infiltration, distant-tumor control, and immune memory	Strong evidence that NP-enhanced relatively low-dose RT can remain immunogenic, but not formal dose exchange because a higher-dose RT-alone isoeffect comparator was not tested.
Zhang et al., 2024 [104]	4T1 breast cancer	R837/BMS@Au8 immune-radioenhancing platform	1 Gy × 3	Quantitative radiosensitization secondary to immune enhancement	D—dose-sparing implied but not directly demonstrated	Strong local and distant immune activation and systemic antitumor effects	Demonstrates immunogenic efficacy at very low per-fraction dose, but low-dose efficacy should not be interpreted as evidence that this schedule substitutes for a higher RT-alone exposure.
Yu et al., 2023 [5]	4T1 TNBC; in vitro and syngeneic in vivo breast-cancer model	Gadolinium oxide nanocrystals (GONs) as MRI-active radiosensitizers	In vitro: 0, 1, 2, 4, and 6 Gy. In vivo: 8 Gy × 1 versus 3 Gy × 3, with or without GONs	Formal clonogenic dose–response analysis with linear-quadratic modeling and SER; quantitative radiosensitization demonstrated in vitro	**C in vitro**—formal clonogenic dose–response/SER evidence; **A in vivo**—same-dose/schedule enhancement without nanoparticle-enabled isoeffect exchange	Increased cGAS–STING/TBK1/IRF3 signaling and intratumoral CD8+ T-cell infiltration; fractionated GON + RT produced the strongest therapeutic and immune response	Direct TNBC evidence linking quantitative nanoparticle radiosensitization with schedule-dependent immune amplification. However, the in vivo comparison altered fractionation and total dose (8 Gy × 1 vs. 3 Gy × 3) and did not test whether a lower NP + RT exposure could reproduce a higher-dose RT-alone tumor isoeffect or preserve systemic lymphocytes.
Peng et al., 2024 [10]	4T1 breast cancer	Ru(II)/MnO2 nanoplatform	4 Gy	Same-dose enhancement	A—same-dose enhancement only	Immune-microenvironment modulation reported	Mechanistically relevant for immune amplification but provides no direct evidence for physical-dose exchange.
Mehrnia et al., 2021 [53]	Breast-cancer cell models	AS1411-targeted gold nanoparticles	Multiple in vitro radiation doses	SER approximately 1.35–1.91 depending on experimental conditions	C—quantitative dose–response radiosensitization; no direct isoeffective lower-dose comparison	Not a principal immune study	Demonstrates dose–response radiosensitization but does not establish in vivo dose exchange.
Lip et al., 2026 [19]	EMT6 and 4T1 breast-cancer models, including syngeneic bilateral 4T1 tumors	Polymer–lipid manganese dioxide nanoparticles (PLMD) designed to reduce hypoxia and enhance RT-induced ICD	In vitro: 2–10 Gy dose–response under relevant oxygen conditions. In vivo: primary tumor irradiated with 10 Gy after PLMD; bilateral model used to assess distant response	Formal clonogenic dose–response/LQ analysis with D50/D90 shifts demonstrating quantitative radiosensitization	C in vitro—formal clonogenic dose–response/isoeffect evidence; A in vivo—same-dose 10 Gy enhancement without dose exchange	Reduced tumor hypoxia; increased calreticulin exposure, HMGB1/ATP release, CD8+ T-cell infiltration and CD86+ macrophages; reduced Tregs/CD163+ macrophages; enhanced distant-tumor control and abscopal response	Strong direct breast evidence connecting quantitative radiosensitization with immune amplification and an abscopal effect. The in vivo study nevertheless used the same 10 Gy RT dose and therefore spent the amplification margin on greater tumor/immune effect rather than demonstrating physical-dose exchange or systemic lymphocyte preservation.
Xu et al., 2026 [20]	4T1 breast cancer	Functionalized self-assembled EGCG nanoparticles combined with FLASH-RT	8-MeV electron FLASH; same FLASH dose in comparator groups	Enhanced ROS generation and DNA damage; no formal isoeffect ratio	A—same-dose enhancement only	Increased splenic DC maturation, B-cell differentiation, CD8+ T cells, NK cells, and memory T-cell responses	Particularly relevant because it directly combines breast cancer, nanoparticles, FLASH, and systemic immune endpoints, but does not demonstrate NP-enabled dose reduction or lymphocyte sparing.
Bonvalot et al., 2019 [55]	Locally advanced soft-tissue sarcoma; randomized clinical trial	Intratumoral hafnium oxide NBTXR3 radioenhancer	50 Gy in 25 fractions in both treatment groups	Improved pathological response with NBTXR3 + RT compared with the same RT prescription alone	A—randomized same-dose clinical radioenhancement; no physical-dose exchange tested	Not a primary clinical immune-endpoint study; immunogenic effects are supported principally by complementary preclinical NBTXR3 studies	Clinical proof that nanoparticle radioenhancement can produce a meaningful biological amplification margin at an unchanged RT prescription, but no evidence that this margin can be exchanged for reduced physical radiation exposure.

Categories describe the type and directness of dose-exchange evidence, not methodological quality. Category C denotes quantitative dose–response or isoeffect evidence but not necessarily demonstrated physical-dose reduction in vivo; a study may meet Category C in vitro while providing only Category A evidence in vivo. Low-dose efficacy, an elevated SER/DEF, or greater tumor response at unchanged prescription should not be interpreted as demonstrated dose exchange. The strongest translational evidence would require a prospectively defined lower-exposure NP + RT regimen to reproduce a prespecified tumor effect achieved with higher-exposure RT alone, preferably in vivo, while also demonstrating reduced immune-relevant exposure and biological injury. Classifications were assigned separately where evidence differed between experimental levels. No study here was classified Category E.

**Table 4:** Complementary radiotherapy strategies relevant to immune preservation and NP-enabled immune-dose shaping.

Study	Strategy/Model	Exposure Variable Modified	Immune/Dosimetric Endpoint	Main Finding	Limitation	Relevance to NP-Enabled Immune-Dose Shaping
Chen et al., 2024 [46]	Reduced-tumor-area irradiation in murine breast cancer	Irradiated tumor volume	Circulating and peritumoral lymphocytes; cGAS–STING; DAMPs; DC activation; systemic antitumor response	Reduced-area RT maintained primary tumor control relative to conventional irradiation while better preserving circulating/peritumoral lymphocytes and retaining immunogenic signaling and systemic effects.	Preclinical model; reduced-volume irradiation cannot be directly translated into omission of clinically indicated target tissue.	Strongest direct breast-specific proof of principle that spatial restriction can preserve immunity without necessarily sacrificing tumor effect.
Mathieu et al., 2023 [47]	50% versus 100% tumor irradiation in orthotopic 67NR breast tumors; additional LLC mechanistic experiments	Spatial distribution/irradiated tumor fraction	STING activation; circulating cytokines; inflammatory response	Partial tumor exposure produced greater STING activation in 67NR tumors and a more proinflammatory cytokine profile than whole-tumor exposure.	Mechanistic preclinical study; does not establish equivalent long-term tumor control or lymphocyte sparing.	Demonstrates that the spatial pattern of radiation exposure itself can alter immunogenic signaling, supporting volume as an immune-dose-shaping dimension rather than merely a physical dosimetric variable.
Cheptea et al., 2025 [45]	Ipsilateral lateral-decubitus versus supine whole-breast RT	Treatment geometry/incidental lymphoid irradiation	EDIC; axillary dose	Lateral positioning approximately halved EDIC and markedly reduced incidental level-I axillary exposure.	Planning study; no measured lymphocyte endpoint; applicable only when nodal irradiation is not indicated.	Geometry lever: demonstrates that treatment setup can reduce both circulating-immune and regional-lymphoid exposure without altering the prescribed target when nodal treatment is unnecessary.
Loap et al., 2024 [43]	IMPT versus VMAT for locoregional breast RT	Modality/integral and distributed low-dose exposure	EDIC; integral dose	IMPT reduced median EDIC from approximately 3.37 to 2.13 Gy, mainly through reduction of integral dose.	Small planning cohort; EDIC is a static surrogate; no direct ALC validation.	Modality lever: illustrates how reducing integral and distributed low-dose exposure could lower the physical immune cost of treatment.
Loap et al., 2025 [44]	DIBH combined with conventional or hypofractionated right-sided locoregional RT	Geometry + fraction number	EDIC	DIBH lowered EDIC, with additional reduction from hypofractionation.	Dosimetric study only; biological immune preservation not demonstrated.	Combined geometry/fractionation lever: shows that separate treatment-design dimensions can produce additive reductions in estimated immune exposure.
Yuan et al., 2018 [78]	Conventional versus hypofractionated postmastectomy RT	Fraction number/repeated blood-pool exposure	Serial lymphocyte counts and recovery	Acute lymphocyte depletion occurred with both schedules, but recovery was more favorable after the shorter regimen.	Fraction number changes together with dose per fraction and total treatment time, preventing isolation of a single causal component.	Fractionation lever: supports the hypothesis that reducing repeated treatment encounters may influence systemic immune injury and recovery.
Sia et al., 2021 [6]	AT3-OVA mammary tumor model with multiple rationally selected RT schedules	Dose per fraction and BED	CD8+ T cells; NK cells; Tregs; anti-PD-1 response; immune memory	Dose per fraction, rather than BED alone, determined CD8+-mediated antitumor activity and anti-PD-1 synergy; NK-cell effects were more BED-dependent, while radiation-induced Treg responses strongly modified immune outcome.	Primarily a tumor-immunology study rather than a lymphocyte-sparing experiment; findings derive principally from one mammary model and have not been independently replicated in a second breast model.	Demonstrates that tumor-control/radiobiological equivalence does not guarantee immune equivalence, an essential constraint on fractionation exchange.
McCullum et al., 2023 [33]	HEDOS-4D dynamic blood-flow modeling in 17 proton-treated HCC patients	Fraction number and beam-on time	Blood DVH; modeled ALC depletion and recovery	Reducing 15 to 5 fractions increased predicted end-of-treatment ALC by ~17%; reducing beam-on time to 1 s increased it by ~27.5%; combining both produced ~60.3% mean predicted benefit.	Small non-breast cohort; computational extrapolation and primarily circulating-blood model.	Establishes fractionation and temporal delivery as separable physical determinants of circulating lymphocyte exposure and suggests their effects may be patient specific.
Ryan et al., 2026 [48]	Human peripheral blood lymphocytes exposed ex vivo to conventional versus UHDR 9-MeV electrons	Dose rate/temporal delivery	Chromosomal aberrations; leukocyte viability; BAX/p53 signaling	UHDR reduced unstable chromosome aberrations by approximately 1.5–2-fold and pooled translocations by ~40–50%, with modestly higher viability and reduced BAX under some conditions.	Ex vivo exposure does not reproduce blood circulation, tumor irradiation, or lymphoid-organ exposure.	Provides direct human-cell evidence that delivery rate can modify lymphocyte biological injury at the same physical dose.
Lu et al., 2026 [49]	FLASH versus conventional RT in 4T1 breast cancer	Dose rate/temporal compression	Tumor response; hepatic oxidative, inflammatory, apoptotic and fibrotic injury	Tumor control was broadly comparable while FLASH reduced several measures of hepatic normal-tissue injury.	Not a direct lymphocyte endpoint; measured FLASH dose was modestly higher, limiting strict isoeffect interpretation.	Breast-specific evidence that temporal delivery can widen the therapeutic window, although direct immune preservation remains unproven.
Xu et al., 2026 [20]	Functionalized EGCG nanoparticles combined with FLASH in 4T1 breast cancer	NP amplification + dose-rate modulation	Splenic DC maturation; B cells; CD8+ T cells; NK cells; memory T cells	BENP + FLASH enhanced tumor damage and multiple systemic immune-cell responses relative to FLASH alone.	No reduced-dose NP arm; no formal tumor isoeffect comparison and no direct demonstration of lymphocyte sparing.	Direct intersection of nanoparticles, breast cancer, FLASH, and systemic immune endpoints, but currently evidence for immune activation rather than NP-enabled immune-dose exchange.
Jin et al., 2020 [32]	Dynamic/distributed immune-organ modeling framework	Blood circulation, fractionation, and distributed lymphoid exposure	Modeled lymphocyte depletion across circulating and noncirculating compartments	Conceptualized the immune system as a distributed organ at risk and modeled repeated irradiation of circulating immune cells across fractions.	Model-based framework requiring biological and clinical validation.	Provides the conceptual basis for moving from conventional organ DVHs toward dynamic immune dosimetry.
de Kermenguy et al., 2026 [51]	Multicompartment UHDR modeling in high-grade glioma	Beam timing, fractionation, blood and lymphoid-organ exposure	Predicted circulating-blood and whole-lymphocyte-pool dose	UHDR markedly reduced direct irradiation of circulating blood, but irradiation of lymphoid structures accounted for a substantial residual component of whole-lymphocyte exposure; fractionation remained important.	Non-breast modeling study; results depend on model assumptions and treatment-site anatomy.	Demonstrates why blood-only sparing is insufficient and supports a multicompartment immune-dose framework.

Studies were selected to illustrate complementary, potentially modifiable dimensions of immune-relevant exposure, not to provide an exhaustive catalogue. Evidence ranges from direct clinical/preclinical breast-cancer studies to mechanistic and computational evidence from other tumor types; indirect evidence defines candidate mechanisms and exposure dimensions rather than breast-specific recommendations. The strategies shown are potential immune-dose-shaping levers—volume/spatial restriction, geometry/modality, distributed low-dose exposure, fractionation, temporal/delivery modification, and multicompartment dosimetry—not established substitutes for standard prescriptions or indicated target coverage. Preserved tumor control does not imply preserved immunogenicity, and reducing EDIC, modeled blood dose, or another single surrogate does not indicate preservation of the circulating or total lymphocyte pool; apparent sparing of one compartment may coexist with unchanged or increased exposure of another. These approaches should therefore be evaluated using tumor-control, immunogenicity, and multicompartment immune endpoints together. Some studies are intentionally cross-listed across tables when they inform more than one analytical axis, such as both nanoparticle dose-exchange evidence and a complementary spatial, fractionation, geometric, or temporal immune-dose-shaping dimension.

**Table 5:** Proposed dual-endpoint experimental framework for nanoparticle-enabled immune-dose shaping.

**Domain**	**Required Comparison or Measurement**	**Preferred Endpoint(s)**	**Criterion Supporting Success**	**Major Confounder/Limitation**
Experimental design	Six-arm comparison: (1) untreated/sham; (2) NP alone; (3) standard RT; (4) NP + standard RT; (5) reduced-exposure RT alone; (6) NP + reduced-exposure RT	Prespecified tumor, immune, dosimetric, and NP-distribution endpoints across relevant arms	The critical comparison—standard RT versus NP + reduced-exposure RT—can be interpreted while separately resolving effects of NP alone, NP radioenhancement, and reduced exposure alone	Fewer arms may confound direct NP immunomodulation, radiosensitization, and true immune sparing
Tumor efficacy/isoeffect	Compare standard RT with NP + reduced-exposure RT using a prespecified isoeffect design	Clonogenic survival and dose–response curves in vitro; tumor-growth delay, local control, recurrence, and survival in vivo	NP + reduced-exposure RT meets a prespecified tumor-isoeffect/equivalence criterion relative to standard RT	Nonsignificant difference does not establish equivalence; heterogeneous NP uptake may obscure true biological effect
Tumor immunogenicity	Compare standard RT with NP + reduced-exposure RT at tumor isoeffect	Cytosolic dsDNA; cGAS–STING/TBK1/IRF3 signaling; type I IFN; calreticulin; ATP/HMGB1; DC maturation; CD8+/Treg/NK composition; antigen-specific responses	Tumor immunogenicity is preserved or enhanced despite reduced physical exposure	Equal tumor control does not imply immune equivalence; dose per fraction, spatial exposure, and sequencing may independently alter immune biology
Circulating immune compartment	Longitudinal sampling before, during, immediately after, and during recovery	ALC; CD4+, CD8+, B, NK, naïve/effector/memory subsets; activation/exhaustion markers; proliferation; DNA injury; functional assays	Reduced loss and/or biological injury with improved recovery at maintained tumor effect	Blood contains only a fraction of the total lymphocyte pool; redistribution may mimic depletion or recovery
Immune phenotype and function	Characterize immune-cell number, phenotype, localization, and function, rather than ALC alone	Flow cytometry; activation/exhaustion/memory markers; cytokine production; antigen-specific proliferation; cytotoxicity/NK function	Quantitative preservation is accompanied by preserved or improved functional immune competence	Cell counts may recover without functional recovery, and functional assays are highly model- and timing-dependent
Tumor-draining and regional lymph nodes	Quantify nodal dose and longitudinal cellular/function changes	TdLN cellularity; CD4+/CD8+/Treg subsets; DC activation; antigen-specific expansion; memory populations	Reduced incidental nodal immune injury where oncologically permissible, without compromising indicated nodal treatment	TdLNs or regional nodes may contain occult/established disease; clinically indicated regional nodal irradiation must not be sacrificed for immune sparing
Marrow-dose/hematopoietic exposure	Compare standard RT with an NP-enabled plan designed to reduce dose to relevant hematopoietic marrow while maintaining tumor isoeffect	Mean marrow dose; V3/V5/V10; sternum/rib/vertebral exposure; active-marrow imaging where feasible; ALC and hematologic recovery	Reduced marrow exposure is accompanied by improved lymphocyte/hematopoietic preservation without compromising tumor efficacy or immunogenicity	Active marrow is spatially heterogeneous; conventional CT bone contours are imperfect surrogates, and marrow sparing may conflict with target coverage depending on disease extent
Circulating-blood dosimetry	Quantify static and, where feasible, dynamic dose to circulating blood	EDIC; mean blood dose; percentage of blood exposed; bDVH; V0.1/V0.5/V1; fraction-specific and cumulative exposure	NP-enabled treatment reduces circulating-blood exposure relative to standard RT while maintaining tumor isoeffect	Static EDIC does not model circulation directly; dynamic models depend on assumptions regarding blood flow, recirculation, repair, and mixing
Distributed low-dose/integral exposure	Compare whole-body and normal-tissue low-dose distributions between regimens	V1, V2, V5; integral dose; ipsilateral-lung V1; relevant whole-body low-dose metrics	Distributed low-dose exposure is reduced at preserved tumor effect	Lower prescription dose does not necessarily reduce low-dose spread and may redistribute rather than eliminate immune exposure
Volume/spatial exchange	Compare standard full-target RT with NP-enabled restricted-volume or spatially modified RT using prespecified tumor-isoeffect criteria	Irradiated tumor volume/fraction; dose to peritumoral tissue and relevant lymphoid structures; local cGAS–STING/cytokine signaling; circulating and peritumoral lymphocyte preservation; tumor control	Reduced irradiated volume or altered spatial exposure decreases immune injury without compromising tumor control or tumor immunogenicity	Spatial restriction must not omit oncologically indicated target tissue; reduced-volume irradiation may itself alter local immune signaling, making physical sparing and immunological modulation difficult to disentangle
Geometry/modality exchange	Compare clinically valid alternative positioning, beam geometry, or modality with standard RT, with and without NP enhancement where appropriate	EDIC; integral dose; low-dose volumes; blood exposure; regional lymphoid dose; marrow dose; tumor coverage and conformity	Alternative geometry/modality reduces systemic or regional immune exposure while maintaining oncologically required target coverage and tumor effect	Dosimetric improvement may differ by laterality, anatomy, target extent, and nodal indication; lower EDIC does not by itself demonstrate biological immune preservation
Fractionation exchange	Compare standard fractionation with an NP-enabled alternative using tumor-isoeffect criteria	Number of fractions; dose per fraction; fraction-specific blood exposure; cumulative immune dose; ALC kinetics; tumor immunogenicity	Fewer/reconfigured fractions reduce cumulative immune injury without compromising tumor control or immunogenicity	Dose per fraction and BED can differentially regulate CD8+, NK-cell, and Treg responses; fewer fractions cannot automatically be assumed immunologically superior
Temporal/delivery exchange	Compare conventional and accelerated/UHDR delivery, with and without NP enhancement where appropriate	Beam-on time; dose rate; interbeam intervals; percentage of circulating blood exposed; lymphocyte DNA injury; lymphoid-compartment exposure	Shorter delivery produces measurable reduction in immune exposure or biological injury at equivalent tumor effect	Sparing circulating blood may not spare lymphocytes residing in lymphoid organs; FLASH-specific biological effects remain incompletely defined
Nanoparticle biodistribution and favorable tumor localization	Quantify NP concentration at irradiation and relevant postadministration time points in tumor and systemic immune compartments	Tumor, blood, spleen, TdLN/other nodes, marrow, liver, lung and other relevant organ concentrations; pharmacokinetics	Favorable tumor-to-immune-compartment radioenhancer ratio at the time of RT	Off-target NP accumulation may radiosensitize immune tissues and counteract the intended benefit of lower physical exposure
Direct nanoparticle immune effects	NP-alone versus untreated control, together with NP + RT versus corresponding RT-alone arms	Lymphocyte viability/proliferation; immune phenotype; cytokine production; DC maturation; NK/T-cell function; genomic injury	Direct NP immunomodulation is characterized and can be distinguished from immune preservation resulting from reduced RT exposure	Beneficial or adverse NP-mediated immune effects may otherwise be incorrectly attributed to radiation-dose exchange
Systemic antitumor immunity	Where appropriate, include untreated distant tumor, rechallenge, or functional tumor-specific assay	Distant-tumor control; tumor-specific killing; IFN-γ response; T-cell clonality; memory; rechallenge protection	Systemic antitumor immunity is preserved or enhanced despite reduced physical immune exposure	Strongly model-dependent; affected by tumor burden, immunotherapy, antigenicity, and timing
Overall immune-dose-shaping success	Integrate tumor, immunogenic, systemic immune, dosimetric, and biodistribution results	Composite interpretation rather than a single surrogate endpoint	Reduced-exposure NP + RT is tumor-isoeffective to standard RT, preserves or enhances tumor immunogenicity, and demonstrably reduces biological injury to relevant circulating, lymphoid, and/or hematopoietic immune compartments	Improvement in one compartment may coexist with injury in another; multicompartment assessment is therefore essential

This framework is proposed in this review as an evidence-informed experimental construct, not an established clinical or regulatory standard, distinguishing conventional radioenhancement from true immune-dose shaping, which requires simultaneous demonstration of five components within the same experimental or analytical framework: **(1)** prespecified tumor isoeffect; **(2)** preserved or enhanced tumor immunogenicity; **(3)** demonstrably reduced systemic or multicompartment immune injury; **(4)** immune-relevant physical or dynamic dosimetry confirming the intended reduction in radiation exposure; and **(5)** nanoparticle biodistribution demonstrating favorable tumor localization relative to immune compartments. Reduction in prescription dose, EDIC, blood dose, or any other single dosimetric surrogate should not by itself be interpreted as evidence of immune preservation. Likewise, apparent benefit in one immune compartment may coexist with unchanged or increased exposure or injury in another, making multicompartment assessment essential. A successful strategy should therefore satisfy all five components concurrently rather than rely on improvement in any single endpoint.

## Data Availability

No new data were created or analyzed in this study. Data sharing is not applicable to this article.

## Supplementary Materials

The following supporting information can be downloaded at https://www.mdpi.com/article/10.3390/ijms27198928/s1.

## Abbreviations

The following abbreviations are used in this manuscript:

ALC	Absolute lymphocyte count
ATP	Adenosine triphosphate
AUC	Area under the curve
AuNP	Gold nanoparticle
bDVH	Blood dose–volume histogram
BED	Biologically effective dose
BMI	Body mass index
cGAS	Cyclic GMP–AMP synthase
cGAS–STING	Cyclic GMP–AMP synthase–stimulator of interferon genes
DAMPs	Damage-associated molecular patterns
DC	Dendritic cell
DEF	Dose enhancement factor
DFS	Disease-free survival
DIBH	Deep-inspiration breath hold
DNA	Deoxyribonucleic acid
DSB	DNA double-strand break
DVH	Dose–volume histogram
EDIC	Effective dose to immune cells
EGCG	Epigallocatechin gallate
ER	Estrogen receptor
FLASH	Ultra-high-dose-rate radiotherapy
HCC	Hepatocellular carcinoma
HMGB1	High-mobility group box 1
ICD	Immunogenic cell death
IFN	Interferon
IMPT	Intensity-modulated proton therapy
NK	Natural killer
NP	Nanoparticle
NSCLC	Non-small-cell lung cancer
PARP	Poly(ADP-ribose) polymerase
PD-1	Programmed cell death protein 1
PD-L1	Programmed death-ligand 1
PLC	Peripheral lymphocyte count
RIL	Radiation-induced lymphopenia
ROS	Reactive oxygen species
RT	Radiotherapy
SBRT	Stereotactic body radiotherapy
SER	Sensitizer enhancement ratio
STING	Stimulator of interferon genes
sTILs	Stromal tumor-infiltrating lymphocytes
TdLN	Tumor-draining lymph node
TILs	Tumor-infiltrating lymphocytes
TLR7	Toll-like receptor 7
TNBC	Triple-negative breast cancer
Treg	Regulatory T cell
UHDR	Ultra-high dose rate
VMAT	Volumetric-modulated arc therapy
