# Supplementary material for: Nanoparticle-Enabled Immune-Dose Shaping in Breast Cancer Radiotherapy: Reconciling Tumor Immunogenicity with Systemic Immune Preservation
Source: Int J Mol Sci. 2026 Oct 8;27(19):8928. doi: 10.3390/ijms27198928 (PMC13666163; doi:10.3390/ijms27198928)
Supplement: Supplementary file 1 [file ijms-27-08928-s001.zip › Supplementary Methods S1.pdf]

Supplementary Methods S1. Literature Search Strategy

Search approach. Literature identification was performed using iterative, topic-targeted searches of PubMed/MEDLINE, Embase, Web of Science, and Scopus, supplemented by backward and forward citation tracking and targeted retrieval of high-relevance studies. The search was designed to support a structured, hypothesis-driven narrative synthesis rather than a systematic review; accordingly, no PRISMA-based selection process or formal risk-of-bias assessment was undertaken, and no publication-date restriction was applied.

Search domains. Searches were organized around five overlapping evidence domains: (1) breast cancer radiation-induced lymphopenia and immune dosimetry; (2) nanoparticle-enhanced breast radiotherapy and associated molecular or immunological mechanisms; (3) nanoparticle-enabled radiosensitization and dose-exchange evidence; (4) complementary immune-sparing radiotherapy strategies, including treatment-volume restriction, particle therapy, positioning, breath hold, fractionation, and ultra-high-dose-rate/FLASH radiotherapy; and (5) relevant cross-tumor evidence addressing blood irradiation, lymphoid or marrow exposure, dose-rate effects, or tumor-draining lymph-node biology when breast-specific evidence was insufficient.

Representative search concepts. Search terms were adapted to the syntax of each database and combined concepts related to breast cancer, radiotherapy, lymphopenia, immune-cell dose, circulating lymphocytes, lymphoid organs, bone marrow, effective dose to immune cells (EDIC), nanoparticles, radiosensitization, radioenhancement, dose enhancement, sensitizer enhancement ratio, dose-response or isoeffect analysis, cGAS–STING, immunogenic cell death, fractionation, partial-volume irradiation, proton therapy, FLASH radiotherapy, blood-dose modeling, and nanoparticle biodistribution.

Study identification and curation. Retrieved articles were evaluated for relevance to the predefined evidence domains and to the central question of whether nanoparticle-generated tumor biological amplification could potentially be exchanged for reduced immune-relevant radiation exposure. Full-text assessment emphasized whether lower-dose NP+RT had been directly compared with higher-dose RT alone and whether tumor-control, immunogenicity, systemic immune, dosimetric, or nanoparticle-biodistribution endpoints were reported. In total, 164 articles were retained, retrieved in full text, and individually reviewed. These studies constitute the complete evidence inventory presented in Supplementary Table S1; the main-text reference list contains a curated subset selected for direct relevance, incremental evidentiary contribution, avoidance of redundant citation, and representative coverage across the predefined evidence domains.

Search date. The literature search was last updated on **1 August 2026**.
